# Paeoniflorin as a candidate disease-modifying therapy for diabetic peripheral neuropathy: mechanisms, exposure challenges, and translational priorities

**DOI:** 10.3389/fphar.2026.1751578

**Published:** 2026-04-13

**Authors:** Yu-Ning Liu, Shuai-Ying Jia, Lin-Song Zhou, Xiao-Jun Tang, Zhi Ming, Yao Su, Jing-Yan Lin

**Affiliations:** Department of Anesthesiology, The Affiliated Hospital of North Sichuan Medical College, Nanchong, China

**Keywords:** anti-inflammatory mechanisms, diabetic peripheral neuropathy, drug delivery systems, neuroprotection, oxidative stress, paeoniflorin

## Abstract

Diabetic peripheral neuropathy (DPN) is the most common chronic complication of diabetes and remains largely treated with symptomatic analgesics (e.g., pregabalin, duloxetine) that do not reverse nerve fiber loss or demyelination, underscoring the need for disease-modifying therapies. Paeoniflorin (PF), a plant-derived monoterpene glycoside metabolite from *Paeonia* spp., shows multitarget activity relevant to DPN pathophysiology in preclinical studies, including activation of Nrf2/HO-1 antioxidant signaling, suppression of TLR4/NF-κB-driven neuroinflammation, support of neurotrophic/repair pathways (e.g., BDNF/TrkB) for axonal regeneration and remyelination, and modulation of microvascular pathways (e.g., HIF-1α/VEGF) linked to endoneurial perfusion. We critically appraise this evidence and highlight key translational constraints: very low oral bioavailability and poor intestinal permeability, extensive presystemic biotransformation (microbiome-mediated hydrolysis and CYP-mediated metabolism) with unresolved “active species” (parent PF versus metabolites), and limited DPN-relevant pharmacokinetics, particularly the lack of peripheral nerve/DRG exposure measurements aligned with pharmacodynamic endpoints. Although formulation and delivery approaches may improve exposure, PF-specific validation in DPN models is currently limited and should be distinguished from platform-level concepts. Finally, because DPN patients frequently experience polypharmacy, a clinically meaningful safety narrative requires systematic assessment of CYP/transporter-mediated drug–drug interaction potential. Priority next steps include integrated PK–PD studies with nerve/DRG distribution, metabolite-resolved exposure–activity linkage, PF-specific delivery validation using disease-modifying endpoints beyond pain behavior, and standardized DDI screening to support trial design.

## 1 Introduction

Diabetic peripheral neuropathy (DPN) is one of the most common chronic complications of diabetes, significantly reducing patients’ quality of life and increasing mortality (Feldman et al., 2019). Current treatment strategies primarily focus on symptom relief, with first-line drugs such as pregabalin and duloxetine partially reducing pain scores but failing to reverse the continuous reduction of epidermal nerve fiber density or repair demyelination, thus not achieving the goal of disease-modifying therapy (Tesfaye et al., 2022). The pathological mechanisms of DPN are complex, and their interactions pose major challenges to treatment, highlighting the urgent need for comprehensive therapeutic strategies that can intervene in the core pathological processes.

Paeoniflorin (PF), a major monoterpene glycoside plant metabolite from the *Paeonia* genus, shows multiple regulatory potentials in diabetic neuropathy. Research indicates that PF can alleviate high-glucose-induced oxidative damage by activating the Nrf2-ARE signaling pathway (Yang et al., 2016; Tian et al., 2021; Hu et al., 2024).

Despite PF’s promising neuroprotective effects, its extremely low oral bioavailability limits its clinical application (Fei et al., 2016). To address these biopharmaceutic constraints, delivery and optimization strategies have been proposed; however, it is important to distinguish PF-specific evidence from platform-level concepts unless directly validated with PF in DPN-relevant models. For example, PLGA-PEG nanoparticles, modified with antibodies or ligands for nasal delivery, can significantly enhance the ability to cross the blood-brain barrier and blood-nerve barrier (Maher et al., 2023). Meanwhile, thermosensitive hydrogels made from chitosan/β-glycerophosphate can form a local sustained-release reservoir around the sciatic nerve, prolonging the drug’s half-life and promoting vascular regeneration (Herron et al., 2021; Rahmanian-Devin et al., 2021). While these approaches illustrate feasible delivery principles, whether they improve PF exposure at peripheral nerve/DRG targets and translate into meaningful DPN outcomes requires PF-specific validation.

This narrative review critically synthesizes the current evidence on PF in DPN, with an emphasis on translational relevance, evidence limitations, and prioritized next-step studies. Specifically, we summarize experimental findings on how PF may intersect with key DPN pathomechanisms, including oxidative stress, neuroinflammation, axonal/myelin injury and repair, and microvascular dysfunction. We then highlight major gaps that currently limit translation (e.g., incomplete DPN-contextualized PK/target exposure, uncertainty regarding the active molecular species after presystemic biotransformation, and polypharmacy-relevant safety/DDI considerations) and outline practical priorities for future work.

## 2 Methods

This narrative review aimed to provide a critical and practice-oriented synthesis of the current evidence on PF in the context of DPN. To identify relevant literature, we searched PubMed/MEDLINE and Web of Science Core Collection from database inception to 30 September 2025. Searches used combinations of controlled vocabulary (e.g., MeSH where available) and free-text terms capturing (i) PF and related descriptors and (ii) DPN and closely related phenotypes (including painful diabetic neuropathy and diabetic polyneuropathy), supplemented by mechanism- and endpoint-related concepts (e.g., oxidative stress, neuroinflammation, Schwann cell/myelination, axonal integrity, microvascular dysfunction, and drug delivery). In addition, reference lists of eligible articles and relevant reviews were hand-searched to improve completeness. This review was prepared with reference to the Four Pillars of Best Practice in Ethnopharmacology Research, with particular attention to transparent reporting of material definition, taxonomic validity where applicable, and critical appraisal of pharmacological evidence.

Eligibility criteria and study selection. Records were screened at title/abstract level, followed by full-text assessment. Studies were included if they (1) investigated PF as a defined/purified plant metabolite (or provided PF-specific evidence within a preparation), (2) addressed DPN or closely related diabetic neuropathy phenotypes *in vitro*, *in vivo*, or clinical settings, and (3) reported outcomes relevant to neuropathy pathophysiology and/or function (e.g., neuroinflammatory and oxidative stress markers, neuronal/glial injury markers, myelin/axon readouts, microvascular endpoints, nerve conduction, intraepidermal nerve fiber density, and/or validated behavioral measures). We excluded non-original publications (reviews, editorials, conference abstracts, patents, book chapters), studies not related to diabetic neuropathy, and purely computational/*in silico* reports without biological validation. Non-English publications were not considered due to resource limitations for reliable assessment.

Data extraction and critical appraisal. For each included study, we extracted key design and reporting elements required to evaluate pharmacological and translational strength: model characteristics (including whether *in vitro*, *in vivo*, or clinical and the relevance to DPN), PF material information (e.g., identity confirmation and purity/source where reported), dose or concentration ranges (including minimal effective dose/concentration when available), route and treatment duration, control conditions (vehicle and positive controls where applicable), and primary endpoints. Importantly, to enable a transparent critical assessment consistent with best practice in ethnopharmacology and pharmacology reviews, we coded the reporting of material definition and chemical characterization items as reported/not reported/not applicable, and we documented common methodological limitations (e.g., absent dose–response testing, inadequate controls, short follow-up, or limited endpoint scope). Evidence was synthesized narratively and weighted according to the completeness of these methodological and reporting features rather than by study count alone. Given the heterogeneity of models and endpoints, a formal systematic review workflow and PRISMA reporting were not adopted; however, the search, selection, and appraisal steps were specified to minimize selection bias and to support reproducible interpretation. In addition, the manuscript was checked against the best-practice recommendations provided by the ConPhyMP/GA online tool (https://ga-online.org/best-practice/) (Heinrich et al., 2022). A structured evidence appraisal table summarizing study design, dose range, controls, duration, material definition, and confidence grading is provided in Supplementary Table S1. Scientific names and authorities were verified against Plants of the World Online (Royal Botanic Gardens, Kew).

## 3 Clinical classification and pathological features of DPN

### 3.1 Epidemiology and disease burden of DPN

DPN is the most common and disabling chronic microvascular complication of diabetes, and its epidemiological status and disease burden have become a global public health challenge. According to the International Diabetes Federation (IDF) 2021 report, the global number of people with diabetes has reached 537 million (Sun et al., 2022), with the prevalence of DPN ranging from 30% to 50% (Iqbal et al., 2018). Moreover, as the duration of diabetes increases, the incidence of DPN shows a significant upward trend—patients with a disease duration of over 25 years have a DPN prevalence of up to 60%–65% (Galiero et al., 2023).

The multiple health damages caused by this condition directly exacerbate the disease burden. Diabetic sensory neuropathy can lead to small fiber symptoms such as burning sensations and prickling, often accompanied by large fiber damage, resulting in the loss of touch or vibration sensation (Pop-Busui et al., 2022). Approximately 50% of DPN patients are affected, and the progression to loss of protective sensation in the feet—detected through monofilament or vibration sense testing—is a core trigger for diabetic foot ulcers (Paisey et al., 2016; Iqbal et al., 2018). The involvement of motor nerves in DPN can manifest as distal muscle weakness and reduced reflexes, leading to gait and balance disorders, significantly increasing the risk of falls and fractures (Beeve et al., 2019; Reeves et al., 2021). Autonomic neuropathy further increases the overall and cardiovascular mortality in diabetic patients through mechanisms such as asymptomatic myocardial ischemia, gastrointestinal paralysis, and bladder dysfunction (Vinik et al., 2003; Gogan et al., 2025). Clinical studies show that 50%–60% of patients with painful DPN require long-term use of analgesics, and approximately 35% of patients reduce their working hours or even lose their ability to work due to pain (Sadosky et al., 2005; Fan et al., 2021).

The multiple medical expenses caused by DPN are burdensome: in the United States, the costs associated with DPN and its complications account for approximately 27% of the direct medical expenses of diabetes (Gordois et al., 2003). The annual medical cost for patients with painful DPN is 2.3 times higher than that of non-DPN patients (approximately $28,000–30,000 vs. $6,600–14,000) (Sadosky et al., 2015). Even more burdensome are the indirect costs caused by chronic pain management, recurrent hospitalizations, and disability, such as loss of labor, which usually far exceed the direct expenditures (Alleman et al., 2015; Bromberg et al., 2024).

### 3.2 Classification based on nerve injury patterns (sensory, motor, and autonomic neuropathy)

**FIGURE 1:**
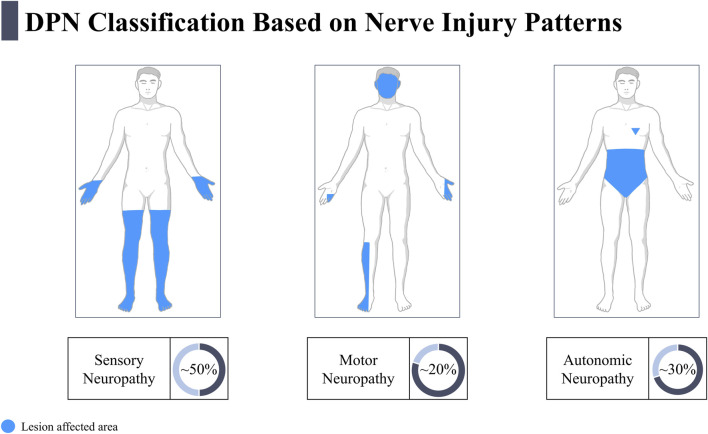
Classification of Diabetic Peripheral Neuropathy Based on Nerve Injury Patterns. This figure shows the three different types of neuropathy: sensory neuropathy affects approximately 50% of patients and primarily manifests as sensory loss in the limbs; motor neuropathy affects about 20% of patients, usually impacting motor functions in the legs; autonomic neuropathy affects around 30% of patients, mainly involving the autonomic nerve function of the heart, gastrointestinal tract, and urogenital system. The blue areas indicate the regions affected by the lesions.

The clinical manifestations of DPN exhibit high heterogeneity. Based on the type of affected nerves and the characteristics of dysfunction, DPN can be categorized into three main subtypes: sensory neuropathy, motor neuropathy, and autonomic neuropathy (Figure 1), each with distinct pathological mechanisms and clinical management strategies.

#### 3.2.1 Sensory neuropathy

Sensory neuropathy is the most common subtype of DPN, primarily affecting small unmyelinated C fibers and thinly myelinated Aδ fibers, presenting with a “sock-glove” distribution (Hoeijmakers et al., 2012). Early in the disease course, patients often experience hyperalgesia and spontaneous burning or tingling pain, which may later progress to the loss of pain and temperature sensation (Farhad, 2019). Pathologically, distal axonal degeneration occurs prior to neuronal cell body damage, and a ≥30% reduction in epidermal nerve fiber density (IENFD) can serve as an early diagnostic marker (Terkelsen et al., 2017). Approximately 20% of patients with sensory neuropathy will progress to painful DPN, which is closely associated with abnormal function of the Nav1.7/1.8 channels in the dorsal root ganglion (DRG), such as upregulation and phosphorylation modification (Wang et al., 2024).

#### 3.2.2 Motor neuropathy

Motor neuropathy (with an incidence of approximately 15%–29%) primarily affects α-motor neurons, leading to distal muscle weakness and atrophy of intrinsic muscles. Typical manifestations include hammer toes and collapsed arches (Bus et al., 2002). Electrophysiologically, a decrease in the compound muscle action potential (CMAP) amplitude of more than 2 mV, along with prolonged distal latency, is more pronounced than sensory nerve slowing (Zhang et al., 2014). Muscle atrophy and deformities increase plantar pressure, which is a major dynamic risk factor for diabetic foot ulcers (Bus et al., 2005).

#### 3.2.3 Autonomic neuropathy

Autonomic neuropathy is the most insidious and fatal subtype of DPN, potentially affecting regulatory systems such as the cardiovascular, gastrointestinal, urinary, and sweat glands. Cardiovascular manifestations often include resting tachycardia and orthostatic hypotension (Vinik et al., 2003); gastrointestinal symptoms include gastroparesis and constipation-diarrhea alternating syndrome (Kempler et al., 2016). It is noteworthy that patients with Cardiac Autonomic Neuropathy are at significantly increased risk of sudden death due to asymptomatic myocardial ischemia and malignant arrhythmias (Serhiyenko and Serhiyenko, 2018), making it a leading cause of DPN-related mortality.

### 3.3 Pathological markers: axonal degeneration, demyelination, and microvascular damage

Current studies consistently indicate that the core pathological manifestations of DPN include: axonal degeneration (Galiero et al., 2023), Schwann cell-mediated demyelination (Chong and Souayah, 2025), and neurovascular microcirculation dysfunction (Zhu et al., 2024), which together constitute the major forms of damage. Superoxide stress and microvascular ischemia not only stimulate axonal degeneration but also impair the ability to repair myelin and nerve structures, driving these pathological mechanisms to intertwine, forming a vicious cycle that accelerates neurodegeneration (Galiero et al., 2023; Zhu et al., 2024).

Hyperglycemia-induced mitochondrial dysfunction leads to excessive ROS generation, damaging microtubule structures and inhibiting axoplasmic transport, which is a key early mechanism of axonal pathology in DPN (Baptista et al., 2019). Furthermore, abnormal neurofilament phosphorylation mediated by ROS further destabilizes the cytoskeleton, resulting in distal “dying-back” axonal degeneration (Baptista et al., 2019). Ultrastructural and electrophysiological studies also show that in DPN patients, mitochondria within axons commonly exhibit swelling and disruption of cristae, with the incidence of cristae rupture significantly higher than normal, and fast/slow axoplasmic transport significantly reduced, impairing the transport of neurotrophic factors (Chowdhury et al., 2011; Yang et al., 2023). The cross-sectional area of large-diameter fiber axons is significantly reduced, which can serve as an early biological marker for detecting pathological changes, often occurring before clinical symptoms appear (Sima and Zhang, 2014).

Demyelination changes in DPN present unique metabolic characteristics: hyperglycemia inhibits Schwann cell pyruvate dehydrogenase activity via the polyol pathway, resulting in a significant decrease in the synthesis of myelin basic protein (MBP) (Hao et al., 2015). Histopathological slices often show segmental thinning of myelin, separation of myelin lamellae, and the formation of “myelin globoid structures” (Llorián-Salvador et al., 2024). Ultrastructural analysis reveals that AGE-modified myelin is more susceptible to degradation by macrophages (with significant infiltration), and is negatively correlated with nerve conduction velocity (Eckersley, 2002). Unlike hereditary demyelinating diseases, demyelination in DPN is primarily caused by “secondary myelin retraction” by Schwann cells after axonal degeneration (Mizisin, 2014).

Microvascular lesions are the underlying pathological drivers of DPN, primarily affecting the neuroendoneurial microcirculation system: hyperglycemia can induce the upregulation of endothelial ICAM-1 expression, activate leukocyte adhesion and oxidative stress, and cause thickening of the nerve microvascular basement membrane (Malik et al., 1993; Chen and Veves, 2023); increased pericyte apoptosis in circulation, along with the loss of capillary tone, leads to a reduction of blood flow in nerve regions by over 35% (Wang et al., 2023). Meanwhile, procoagulant mechanisms like PAF trigger thrombosis and fibrin deposition, causing nerve tissue hypoxia (pO_2_ ≤ 15 mmHg) (Wang et al., 2023). These microcirculatory disorders present as “no-reflow phenomena,” with occlusion rates of nourishing vessels in the feet reaching over 40% (Malik et al., 1993; Chen and Veves, 2023). Furthermore, exosome-mediated miRNAs, such as miR-128, exacerbate nerve ischemic damage by inhibiting HIF-1α and downregulating angiogenesis mechanisms—this effect is more pronounced in patients with a disease duration of over 10 years (Tajabadi et al., 2025).

## 4 Core pathophysiological mechanisms of DPN

**FIGURE 2:**
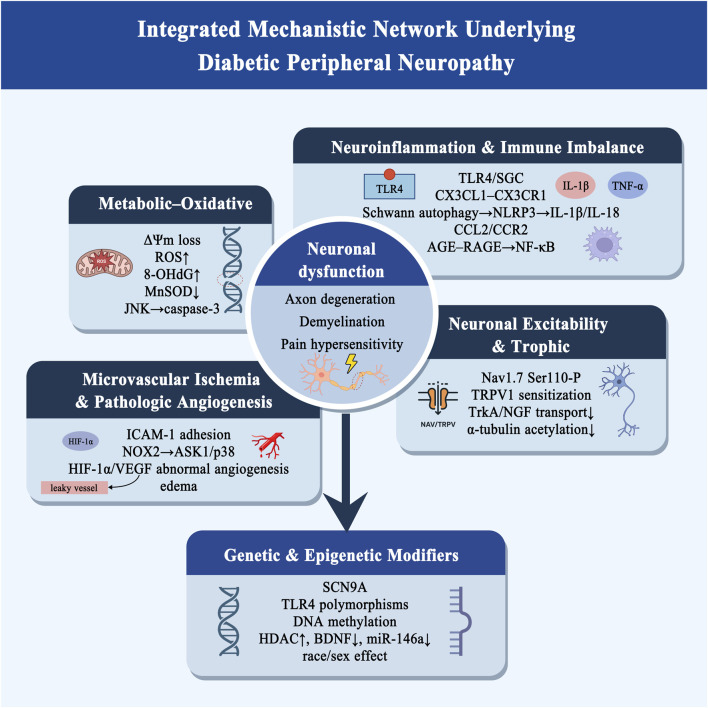
Integrated Mechanistic Network Underlying Diabetic Peripheral Neuropathy. The figure illustrates an integrated pathogenic network of diabetic peripheral neuropathy. The central module, neuronal dysfunction, represents the convergent outcome characterized by axonal degeneration, demyelination, and pain hypersensitivity. (i) Metabolic–Oxidative Node: Hyperglycemia disrupts mitochondrial membrane potential (ΔΨm), impairs the electron transport chain, and elevates reactive oxygen species (ROS). These changes cause mtDNA damage (↑8-OHdG), reduce MnSOD activity, and activate the JNK–caspase-3 apoptotic cascade. AGE–RAGE interaction enhances NF-κB–dependent inflammation and damages the blood–nerve barrier by inhibiting VEGFR2 phosphorylation and degrading occludin. (ii) Neuroinflammation and Immune Imbalance: Sustained TLR4 activation in satellite glial cells and autophagy impairment in Schwann cells activate the NLRP3/ASC/caspase-1 inflammasome, increasing IL-1β, IL-18, and TNF-α. CCL2/CCR2-dependent macrophage infiltration (TREM2^+^CD68^+^, M1 phenotype) and AGE–RAGE–NF-κB signaling amplify neuroinflammation and tissue injury. (iii) Microvascular Ischemia and Pathologic Angiogenesis: ICAM-1–mediated leukocyte adhesion, NOX2-derived ROS, and ASK1/p38MAPK activation induce endothelial apoptosis and ischemia. HIF-1α–dependent VEGF overexpression promotes leaky and disorganized neovascularization, leading to edema and secondary axonal compression. (iv) Neuronal Excitability and Trophic Node: PKC/p38 activation increases Nav1. 7 Ser110 phosphorylation and persistent Na^+^ influx, while AGE–RAGE–ROS–PKC–Src signaling sensitizes TRPV1 channels. NGF–TrkA endocytosis and retrograde transport are reduced due to α-tubulin deacetylation, forming a feedback loop that reinforces oxidative stress and excitotoxicity. Genetic and Epigenetic Modifiers: Variants in SCN9A and TLR4, altered DNA methylation, HDAC upregulation, reduced BDNF, and miR-146a downregulation contribute to interindividual differences in DPN susceptibility, phenotype, and treatment response. Abbreviation: SGC, satellite glial cell.

DPN results from the convergence of metabolic, inflammatory, vascular, and neuronal disturbances driven by chronic hyperglycemia. Mitochondrial oxidative stress, immune activation, microvascular ischemia, and impaired neuronal excitability collectively lead to axonal degeneration, demyelination, and pain hypersensitivity. Genetic and epigenetic modifiers, including *SCN9A* and *TLR4* variants, histone deacetylation, and dysregulated miRNAs, further shape disease susceptibility and phenotypic diversity. These interconnected processes establish a multidimensional pathogenic network that drives both the onset and progression of DPN (Figure 2).

### 4.1 Metabolic dysregulation driving mechanisms

Hyperglycemia-induced metabolic dysregulation disrupts mitochondrial membrane potential, leading to electron transport chain dysfunction and increased ROS generation (Hedayat et al., 2023). These oxidative damages result in mtDNA injury (such as elevated 8-OHdG levels), decreased antioxidant enzyme function (e.g., MnSOD), and hindered axonal transport. Studies in rodents and DRG cells have shown that this process activates the JNK-caspase-3 pathway, triggering neuronal apoptosis (Schmeichel et al., 2003; Wang et al., 2023).

Under prolonged hyperglycemic conditions, proteins undergo non-enzymatic glycosylation, forming Schiff bases, Amadori intermediates, which are further oxidized to form advanced glycation end products (AGEs). The expression of AGEs and their receptor RAGE is significantly elevated in the serum and peripheral nerves of DPN patients (Yan et al., 2009). AGEs bind to RAGE, initiating IKKβ activation and IκB degradation, causing NF-κB translocation to the nucleus and upregulation of inflammatory cytokines such as TNF-α and IL-6 (Osorio et al., 2016; Zhou et al., 2024). Additionally, this axis, when excessively activated in endothelial cells, suppresses VEGFR2 phosphorylation and induces degradation of tight junction proteins (such as occludin), compromising the blood-nerve barrier (Stirban et al., 2014).

Recent studies have highlighted the critical role of mitochondrial biogenesis in neurogenesis and neuronal survival. As shown by Bai et al. (Bai et al., 2025), the induction of mitochondrial biogenesis following ischemic injury significantly promotes neurogenesis and cognitive recovery by enhancing the function of neural stem cells in the hippocampus. This concept resonates with paeoniflorin’s neuroprotective effect, where enhancing mitochondrial function could help counteract neurodegenerative processes observed in diabetic peripheral neuropathy.

### 4.2 Neuroinflammation and immune microenvironment imbalance

The progression of DPN is closely associated with an imbalance in the neuroimmune microenvironment. The core mechanisms include: sustained hyperglycemia activating TLR4 on DRG satellite glial cells (SGCs), accompanied by a significant increase in GFAP expression (Liu Y. et al., 2025), which initiates coupling and enhanced inflammation between SGCs and neurons via the CX3CL1/CX3CR1 signaling axis (Chen et al., 2017; Zang et al., 2023; Hernandez-Reyes and Oo, 2025); activated SGCs secrete IL-1β and TNF-α (Zhang D. et al., 2023), inducing abnormal neurofilament phosphorylation and blocking axonal transport (Hanani and Spray, 2020; Derouiche, 2022); at the same time, Schwann cell autophagy is inhibited, leading to ROS accumulation, which activates the NLRP3/ASC/caspase-1 inflammasome, driving the production of mature IL-1β and IL-18 (Cheng et al., 2020; Abd Razak et al., 2024). Clinical evidence also shows that IL-18 levels are positively correlated with the severity of painful neuropathy, validating the key role of inflammatory mediators in nerve injury progression (Baka et al., 2021).

TRPV1 plays a crucial role in inflammatory diseases, including sepsis-associated encephalopathy, where its activation suppresses pyroptosis and subsequent neuronal damage. Activation of TRPV1 with CAP has been observed to reduce inflammatory cytokines like IL-1β and IL-18, thus offering a neuroprotective effect in animal models (Long et al., 2025).

Immune cell infiltration and the neuroischemia mechanism constitute the second stage of neuroendoneurial injury. MCP-1 (CCL2) drives CCR2^+^ monocyte/macrophage infiltration into the nerve epineurium (Nashtahosseini et al., 2025). These infiltrating cells predominantly exhibit an M1 phenotype, especially TREM2^+^CD68^+^ macrophage subgroups, which secrete TNF-α, IL-6, and high levels of MMP-9, aggravating the inflammatory “storm” and disrupting the axonal guidance microenvironment (Candelario-Jalil et al., 2007). Meanwhile, NOX2 activation causes ROS (mainly superoxide anions) accumulation in the neuroendoneurium, where ROS acts as a second messenger to activate the ASK1/p38MAPK pathway, inducing apoptosis in peripheral nerve endothelial and neuronal cells (Watanabe et al., 2015). Additionally, infiltrating mast cells release histamine and platelet-activating factor (PAF), which induce upregulation of ICAM-1 expression in the nerve fascicle microvascular endothelium, enhancing leukocyte adhesion and causing microcirculatory blockages (Thacker et al., 2009; Kajiwara et al., 2010). Overall, this “immune infiltration-inflammation storm-oxidation-microcirculatory obstruction” three-stage mechanism constitutes the core pathway for neuroendoneurial injury in DPN and offers potential strategies for targeted intervention.

This immune-vascular interaction ultimately leads to severe neuroendoneurial hypoxia (Low et al., 1989), activating hypoxia-inducible factor 1α (HIF-1α) and driving VEGF-A overexpression (Catrina and Zheng, 2021; Ding et al., 2023). However, the pathological angiogenesis forms disordered capillary loops with increased permeability, causing plasma protein leakage and nerve edema, which further compresses nerve fibers and worsens ischemia (Fang et al., 2018).

### 4.3 Neuronal structural and functional damage

The core manifestation of neuronal damage in DPN is a dual pathological process involving ion channel dysfunction and the loss of neurotrophic signaling. In sensory neurons, the voltage-gated sodium channel Nav1.7 is a key target for regulating pain transmission in DRG neurons. Hyperglycemia activates the PKC and p38 MAPK pathways, leading to increased phosphorylation at the Ser110 site on the N-terminus of Nav1.7, which promotes channel membrane expression and enhances persistent sodium influx (Tyagi et al., 2024). Clinical studies have confirmed a positive correlation between Nav1.7 channel density in skin nerve biopsies and pain scores in DPN patients (Albrecht et al., 2021). Additionally, the AGEs-RAGE axis abnormally activates TRPV1 channel function sensitization via the PLC-PKC pathway, independent of channel expression upregulation. Studies have shown that under high glucose conditions, CAP-induced TRPV1 currents in DRG neurons are enhanced, and this effect depends on RAGE, ROS, PKC, and Src kinase activity, with no significant change in channel expression levels (Lam et al., 2018). In DPN animal models, the proportion of TRPV1^+^ DRG neurons is significantly increased (Chen et al., 2019). Channel sensitization leads to increased excitability to thermal stimuli, triggering thermal hyperalgesia (Gao et al., 2024). This mechanism provides a molecular basis for the markedly enhanced pain response to thermal stimuli.

Disruption of neurotrophic factor signaling has been confirmed as one of the key mechanisms driving neuronal degenerative changes in diabetic neuropathy. Hyperglycemia can disrupt neurotrophic support through multiple pathways. First, in retrograde transport of nerve growth factor (NGF), diabetes significantly inhibits phosphorylation of the tyrosine kinase domain of the TrkA receptor, decreasing phosphorylation efficiency, and thus reducing the internalization rate of the NGF-TrkA complex (Baptista et al., 2019; Yang et al., 2023). Furthermore, diabetes induces a reduction in α-tubulin acetylation, and this loss of acetylation directly impacts microtubule stability and axoplasmic transport efficiency, significantly decreasing the rate of NGF transport in axons and ultimately reducing NGF concentrations in the DRG neuronal cell bodies (Middlemas et al., 2003; Chandra et al., 2024). These multi-dimensional disruptions in neurotrophic signaling may be a core mechanism driving the continuous exacerbation of neuronal degeneration.

Sustained hyperglycemia does not act in isolation to affect ion channels or neurotrophic factors but rather forms a positive feedback loop through shared signaling nodes. On one hand, overactivated TRPV1 and Nav1.7 lead to sustained sodium and calcium influx, triggering mitochondrial calcium overload and ROS bursts (Yang et al., 2023). On the other hand, ROS and the RAGE-NF-κB axis downregulate the phosphorylation of the tyrosine kinase domain of TrkA, inhibiting NGF internalization and retrograde transport (Baptista et al., 2019), leading to reduced NGF concentrations in the cell body, which in turn weakens the suppression of transcription factors (such as REST) that negatively regulate Nav1.7 and TRPV1 expression (Yang et al., 2023; Tyagi et al., 2024). Thus, ion channel abnormalities and the loss of neurotrophic signaling mutually reinforce each other in the high glucose environment, collectively driving neuronal excitability imbalance and degenerative changes.

### 4.4 Genetic and epigenetic factors in individual differences

In addition to hyperglycemia-induced metabolic dysregulation, genetic and epigenetic factors also drive the susceptibility, progression, and therapeutic response variability of DPN, constituting a key source of clinical heterogeneity (Singh et al., 2013; Li et al., 2015; Blesneac et al., 2018). In human genetic evidence, variations in SCN9A (encoding Nav1.7) are closely associated with pain phenotypes: the common functional variant rs6746030 is linked to changes in pain threshold, and sequencing studies targeting DPN further reveal that rare functional variants of Nav1.7 are significantly enriched in painful DPN patients, suggesting the involvement of peripheral excitatory channel genetic backgrounds in the formation of pain phenotypes (Li et al., 2015; Blesneac et al., 2018). In innate immunity pathways, polymorphisms in the TLR4 gene (such as rs4986790, Asp299Gly) show variable associations with the risk of diabetic complications in different populations: in a North Indian T2D cohort, it is associated with an increased risk of foot ulcers, while in a German T2D cohort, it is reported to be linked to a decreased prevalence of neuropathy, suggesting that genetic differences in the TLR4 signaling axis may influence DPN outcomes by modulating inflammatory sensitivity (Rudofsky et al., 2004; Singh et al., 2013). At the epigenetic level, whole-genome methylation studies from two independent cohorts have identified methylation characteristics and candidate gene loci that differentiate painful and painless DPN.

In rodent DPN models, inhibition of inflammatory genes improves the neurological phenotype, and in human studies, such genes are associated with DPN occurrence, suggesting that the “genetic-epigenetic-inflammation/neural excitability” framework is a reasonable explanation for the heterogeneity of painful DPN (Liu et al., 2017; Guo et al., 2020; Kwiatkowska et al., 2025).

Epigenetic regulation amplifies the individual differences in DPN. High glucose environments can induce the upregulation of deacetylase expression, especially increasing the activity of HDAC1, thereby altering histone acetylation states in peripheral nerve support cells and inhibiting autophagy and neuroprotective pathways. Numerous animal and cell studies have shown that HDAC upregulation is associated with diabetic-related neurodegeneration and pain phenotypes, and HDAC inhibitors can reverse some pathological changes and alleviate pain (Du et al., 2019; Thakur et al., 2024).

In the peripheral nervous system, decreased BDNF levels are associated with reduced neuronal function and regenerative ability. Both human and animal studies have reported that BDNF levels are lower in DPN patients or diabetic models compared to those without neuropathy, and BDNF supplementation can improve DRG neuronal excitability and promote axonal regeneration (Krabbe et al., 2007; Li et al., 2016).

The miRNA network also participates in this process. miR-146a is commonly downregulated under high glucose conditions, leading to reduced inhibition of target genes such as IRAK1/TRAF6, thereby amplifying the expression of NF-κB downstream inflammatory cytokines (e.g., IL-6, TNF-α) and intensifying the local inflammatory microenvironment. Molecular data from both animal and human samples support the key role of miR-146a in DPN inflammation regulation (Feng et al., 2018).

Ethnic and gender differences can modulate the effects of the above genetic-epigenetic networks. For example, the common functional SNP rs6746030 of SCN9A (encoding Nav1.7) shows distinct allele frequency variations across geographical and ethnic groups and is associated with pain threshold and pain phenotypes, which may partly explain the heterogeneity of drug (including analgesics or PF-containing treatments) efficacy in different populations (Estacion et al., 2009).

## 5 Limitations and unmet needs in current DPN treatments

### 5.1 Efficacy bottleneck of first-line medications

Currently, the primary pharmacological interventions for DPN remain gabapentinoids (such as gabapentin and pregabalin) and serotonin-norepinephrine reuptake inhibitors (SNRIs, such as duloxetine) as first-line treatment options. These drugs primarily work by targeting the α2δ subunit of voltage-gated calcium channels (VGCC), inhibiting central sensitization and peripheral pain transmission (Jang and Oh, 2023; Zhang Y. et al., 2024). Although some short-term pain relief has been achieved, their overall efficacy remains limited.

Several randomized controlled trials have shown that gabapentinoids provide ≥50% pain relief in only 30%–50% of patients with DPN-related pain (Richter et al., 2005; Freeman et al., 2008), with efficacy highly dependent on the dosage. Notably, after 12 weeks of continuous treatment, the response rate drops to about 42% of the initial treatment level, suggesting the presence of drug tolerance or diminishing efficacy over time (Javed et al., 2018; Gordon et al., 2022). Additionally, SNRIs such as duloxetine exert their analgesic effect by enhancing serotonin (5-HT) and norepinephrine (NE)-mediated descending inhibitory systems in the spinal cord (Obata, 2017). However, the actual clinical efficacy remains limited. In a Phase III clinical study, at the recommended dose of 60 mg/day, the discontinuation rate due to adverse effects was 13.2%, and about 49% of DPN patients achieved ≥50% pain relief, compared to 26% in the placebo group (Goldstein et al., 2005). Overall, while duloxetine has some role in managing neuropathic pain, its limited analgesic efficacy, inadequate pain spectrum coverage, and side-effect burden indicate that it fails to meet the practical clinical needs for DPN treatment.

Despite the partial effectiveness of gabapentinoids (such as gabapentin and pregabalin) and duloxetine in symptomatic treatment of DPN-related pain, these medications still have key limitations in terms of nerve structure repair and functional recovery. First, these drugs fail to significantly intervene in axonal degeneration and show no marked improvement in large fiber function or nerve conduction (Chiang et al., 2022). Second, studies show that they do not reverse small fiber damage (Javed et al., 2018; Calcutt, 2020). A study on patients with type 2 diabetes and peripheral neuropathy found that after 3 months of gabapentin treatment, patients’ HRV parameters such as SDNN and HF showed significant improvement, suggesting a positive effect on cardiac autonomic function, but the long-term decline in autonomic function could not be completely reversed (Ermis et al., 2010). In contrast, studies on duloxetine’s effect on HRV are limited, with existing systematic reviews mainly focusing on its pain-relief effects and not showing significant effects in preventing HRV decline.

In summary, although current first-line medications can alleviate DPN pain to some extent, their efficacy is limited, coverage is insufficient, and they lack functional repair effects. These drugs fall short of meeting the clinical needs for pain control and nerve function maintenance in DPN patients.

### 5.2 Adverse effect profiles of medications

While the efficacy of current first-line medications in the treatment of DPN has been affirmed, safety concerns have increasingly emerged, limiting their long-term use. For example, gabapentinoids exhibit a high affinity for the α2δ subunit of VGCC. While they effectively inhibit presynaptic vesicle release and relieve neuropathic pain, they also frequently induce central side effects, with dizziness and drowsiness being the most common (Kato et al., 2015; Ohishi et al., 2015).

A meta-analysis further indicated that about 30% of patients discontinued pregabalin treatment due to central side effects such as drowsiness and dizziness, significantly impacting patient compliance (Quilici et al., 2009). Mechanistically, gabapentinoids (such as gabapentin) specifically bind to the α2δ subunit of high-voltage-activated calcium channels (HVA), inhibiting the activity of presynaptic P/Q-type calcium channels and reducing the release of excitatory neurotransmitters (such as glutamate), thereby broadly lowering neuronal excitability. This effect is particularly prominent in thalamic relay nuclei (such as the ventral basal ganglia) and can interfere with thalamocortical sensory information transmission, leading to functional inhibition in cognitive domains such as attention and executive function (Holtkamp and Meierkord, 2007). Notably, gabapentin does not directly act on low-voltage-activated T-type calcium channels (Cav3. 1-Cav3. 3). T-type channels regulate δ-oscillations (0.5–4 Hz) via the “window current” mechanism, which plays a key role in thalamocortical rhythm synchronization (such as the sleep-wake transition), with dysfunction linked to cognitive symptoms in diseases such as epilepsy and sleep disorders (Holtkamp and Meierkord, 2007). Gabapentin-associated psychomotor slowing (e.g., thought latency, slower reaction time) primarily stems from reduced synaptic efficacy caused by HVA channel inhibition, rather than specific modulation of rhythmic pathways (Holtkamp and Meierkord, 2007).

In contrast, while duloxetine (an SNRI) provides similar pain relief to pregabalin, its cardiovascular safety issues have raised concerns. A systematic review and meta-analysis encompassing 17 randomized controlled trials (dosing range 30–120 mg/day, with a follow-up duration of up to 13 weeks) showed that duloxetine increased the average heart rate by approximately 2.22 bpm (95% CI: 1.53–2.91) (Park et al., 2020). Moreover, case reports have indicated that duloxetine may cause QTc interval prolongation and potentially fatal arrhythmias (Štuhec, 2013). Its metabolic pathway also increases the risk of drug interactions. Duloxetine, as a moderate CYP2D6 inhibitor, significantly inhibits the metabolism of β-blockers such as metoprolol (2022).

In conclusion, although gabapentinoids and duloxetine are still widely used as first-line treatments for DPN, the adverse effects associated with their mechanisms of action have become key factors limiting their long-term clinical application. Therefore, from molecular target selection to mechanism optimization, how to maximize the reduction of central and cardiovascular side effects while maintaining analgesic efficacy has become a core challenge in current DPN drug development. Identifying novel analgesic targets and intervention strategies that balance efficacy and safety, particularly those that bypass traditional neurotransmitter pathways and reduce systemic load, is a crucial direction for future breakthroughs in this field.

### 5.3 Current lack of disease-modifying therapies (DMTs)

The most pressing challenge in the current treatment landscape of DPN is the systemic absence of disease-modifying therapies (DMTs). Existing medications can only alleviate symptoms but fail to intervene in the core pathological mechanisms of neurodegenerative processes. This gap is reflected in three major aspects.

#### 5.3.1 Axonal regeneration deficiency

In DPN, chronic hyperglycemia continuously activates the RhoA/ROCK signaling pathway (Zhou et al., 2018), leading to the excessive phosphorylation of collapsin response mediator protein 2 (CRMP2), a protein involved in growth cone collapse. The phosphorylation level of CRMP2 is significantly higher than in controls, severely inhibiting the regeneration capacity of neuronal axons (Arimura et al., 2005). Even in patients with optimized blood glucose control (HbA1c <7.0%), clinical studies have shown that their sural nerve biopsies reveal lower nerve regeneration cluster density than that of pre-diabetic levels, indicating that simple blood sugar control is insufficient to reverse the pathological degeneration of DPN (Hur et al., 2013).

More concerningly, although neurotrophic factors such as nerve growth factor (NGF) and glial cell line-derived neurotrophic factor (GDNF) have shown strong neuroprotective and reparative potential, they have widely failed in Phase II clinical trials, primarily because they cannot effectively cross the blood-nerve barrier, leading to a delivery efficiency of less than 15% in dorsal root ganglion (DRG) neurons (Apfel, 2002; Griffin et al., 2020). This underscores the need for effective targeting and delivery of neurotrophic factors, which remains a critical bottleneck in DPN treatment strategies.

#### 5.3.2 Failure of myelin repair

Myelin repair failure is one of the key mechanisms driving the progression of DPN. The core pathology lies in the impaired remyelination capacity of Schwann cells. In a hyperglycemic environment, the Neuregulin 1/ErbB2 signaling axis malfunctions, with abnormal activation of the ErbB2 receptor tyrosine kinase and concomitant impairment of Schwann cell proliferation and differentiation, hindering myelin regeneration (Pan and Dobrowsky, 2013). While this pathway plays a crucial role in maintaining myelin integrity, no effective drug interventions have been developed to “activate” the Neuregulin-1/ErbB2 pathway to promote myelin reconstruction. In contrast, some studies have found that ErbB2 is abnormally phosphorylated in a diabetic state, and the application of small molecule inhibitors (e.g., PKI 166 or erlotinib) can reverse nerve conduction dysfunction, suggesting that the role of this pathway in DPN may exhibit complex bidirectional regulation depending on the stage and location (McGuire et al., 2009).

Additionally, hyperglycemia-induced overexpression of histone deacetylase 6 (HDAC6) leads to a decrease in the acetylation level of the microtubule protein α-tubulin, thereby inhibiting Schwann cell-mediated myelination processes, including myelin sheath wrapping and maintenance (Li et al., 2022; Raouf, 2024). HDAC6 overactivation not only destabilizes microtubules but also disrupts the signal integration between axons and Schwann cells, causing persistent hindrance to peripheral nerve remyelination (Prior et al., 2018). Although non-selective HDAC inhibitors (such as valproate or TSA) have been shown to increase α-tubulin acetylation levels in animal models, improving Schwann cell myelin regeneration and axonal support function (Picci et al., 2020), their broad inhibition of HDAC I/II/IV family members, including neuroprotective HDAC4, often results in severe central side effects such as cognitive dysfunction and motor coordination disorders (Langley et al., 2005; Bahl and Seto, 2021), limiting their clinical application. Therefore, the lack of highly selective and well-tolerated HDAC6 inhibitors has become a major bottleneck hindering progress in DPN treatment.

#### 5.3.3 Lack of microvascular protection

In diabetic neuropathy (DPN), failure in the repair of neurotrophic blood vessels is one of the key disabling mechanisms, with resistance to vascular endothelial growth factor (VEGF) considered the main cause of missing microvascular protection. In diabetic microvascular complications, sFlt-1 binds VEGF with high affinity, blocking its interaction with VEGFR-2 (Di Marco et al., 2009), especially under ischemic stress. In diabetic tissues, the expression of sFlt-1 can increase up to 2.7 times that of non-diabetic tissues, severely impairing angiogenic signaling (Hazarika et al., 2007). This mechanism may play a key role in the failure of neurotrophic blood vessel repair in DPN, presenting a novel target for treatment.

Research has shown that VEGF165, as a key pro-angiogenic factor, has been shown to induce endothelial cell proliferation and lumen formation in diabetic retinopathy models, exhibiting angiogenic activity. However, whether this mechanism can extend to the repair of neurotrophic blood vessels in DPN models remains to be further validated. Moreover, studies also suggest that the increased permeability of newly formed vessels could lead to neuroendoneurial edema, exacerbating inflammation and ischemia, and reducing nerve conduction velocity, indicating that single-agent VEGF treatment may do more harm than good due to abnormal vascular leakage (Taiana et al., 2014; Wazan et al., 2024).

#### 5.3.4 Preliminary exploration and continued lack of emerging therapies

In recent years, several “disease-modifying” or advanced intervention strategies have emerged in the DPN field, but evidence remains insufficient to prove that they can fully reverse axonal damage or myelin loss.

Neuromodulation techniques like spinal cord stimulation (SCS) have been shown to significantly reduce pain scores in painful DPN and have received regulatory approval for expanded indications, but their action is primarily focused on pain modulation rather than promoting axonal regeneration or myelin repair. Additionally, SCS is an invasive treatment, and its cost and long-term follow-up data are still needed to assess sustained efficacy and complication risks (Yeung et al., 2024). Non-invasive electrical stimulation methods, such as FREMS (Frequency Rhythmic Electrical Modulated System), have shown short-term pain relief and improved neurophysiological parameters in several randomized controlled trials and cohort studies, but these studies are mostly small sample or short-term follow-up, making them difficult to serve as a substitute for disease-modifying therapies (Crasto et al., 2022; Gorczyca-Siudak and Dziemidok, 2022). Biological agents targeting inflammatory pathways (e.g., TNF-α or IL-6 pathway inhibitors) have been shown to reduce pro-inflammatory macrophage infiltration and alleviate tissue inflammation in animal models, but human trial data is extremely limited, and long-term suppression of inflammation poses risks of immunosuppression and infection, making it difficult to provide comprehensive protection for microvascular and neuronal structures (Wu et al., 2018; Sen et al., 2023). Stem cell therapies (including mesenchymal stem cells) have shown improvements in nerve conduction and local blood flow in several preclinical and small clinical studies, promoting some degree of sensory and electrophysiological recovery, but face translational barriers such as transplantation sources, immunocompatibility, administration routes, and quality control (standardization), with no large-scale randomized controlled trials confirming long-term benefits (Alizadeh et al., 2024; Alizadeh et al., 2025). New small molecules, such as selective sodium channel modulators like suzetrigine/VX-548, have shown analgesic potential in recent clinical trials and have entered later-stage trials, but their focus remains on symptom management rather than the reversal of pathological axonal degeneration (Study Details | NCT05660538 | Evaluation of Efficacy and Safety of VX-548 for Painful Diabetic Peripheral Neuropathy (DPN) | ClinicalTrials. gov).

Overall, these emerging methods each have their advantages, but the evidence and mechanisms are often single or partially regulated pathways, failing to cover the multifactorial pathological network of DPN. This highlights the research value of multi-target or combination strategies (e.g., combining the multi-target pharmacology of PF with precise delivery/biomaterials).

## 6 Overview of PF and pharmacokinetic properties

### 6.1 Botanical source: an active monoterpene glycoside from paeoniaceae

PF is a monoterpene glycoside metabolite predominantly isolated from species of the genus *Paeonia*, particularly *Paeonia lactiflora* Pall. and *Paeonia veitchii* Lynch (family Paeoniaceae). The taxonomic validity of these species follows accepted botanical nomenclature standards (Plants of the World Online, Kew).

The present review focuses exclusively on purified paeoniflorin as a chemically defined small molecule, rather than crude botanical drugs or multi-component traditional preparations.

PF has the molecular formula C23H28O11 and a molecular weight of 480.45 g/mol. It is registered under CAS No. 23180-57-6 and PubChem CID 442534. Structurally, PF is characterized as a pinane-type monoterpene glycoside containing multiple hydroxyl groups and an ester linkage, contributing to its high polarity and limited membrane permeability. PF is relatively stable under acidic conditions (pH 2–6) but undergoes degradation under alkaline conditions and elevated temperatures.

### 6.2 Toxicology and drug–drug interaction considerations of paeoniflorin

Published toxicological studies indicate that PF exhibits an overall favorable baseline safety profile in preclinical settings (Fan et al., 2012). In rodents, the oral median lethal dose (LD50) is notably high (14.55 g/kg in rats), suggesting a wide safety margin. Even at high oral doses (e.g., 3 g/kg), no significant adverse effects or mortality were observed (Ou et al., 2025). Subacute and chronic toxicity studies further suggest good tolerability across commonly tested ranges (100–400 mg/kg), with no consistent evidence of overt injury to major organs such as liver and kidney (Ha et al., 2011; Jeong et al., 2014). Long-term administration similarly did not produce clear functional alterations or histopathological abnormalities in vital organs, although further surveillance of long-term risks (e.g., reproductive toxicity and carcinogenicity) has been recommended (Hong et al., 2022). Genotoxicity and mutagenicity assays have generally been negative, suggesting no significant gene mutations or chromosomal aberrations under tested conditions (Liu et al., 2006). Safety pharmacology studies also reported no major adverse findings in cardiovascular, central nervous, or urinary systems within the investigated settings (Jiao et al., 2021).

However, DPN patients are frequently older and exposed to polypharmacy, which makes pharmacokinetic drug–drug interactions (DDIs) a clinically relevant translational consideration (Cakir, 2025). From a small-molecule development perspective, DDI liabilities are primarily mediated by modulation of (i) drug-metabolizing enzymes (notably CYPs) and/or (ii) drug transporters (e.g., P-gp/ABCB1 and BCRP/ABCG2) (Huang et al., 2010). Because robust, PF-specific DDI datasets are not yet consolidated, a conservative development-oriented approach would include screening against major CYP isoforms (CYP3A4/5, CYP2D6, CYP2C9, CYP2C19, CYP1A2, and CYP2B6) and key transporters (P-gp/ABCB1, BCRP/ABCG2, OATP1B1/1B3, OAT1/3, OCT2, and MATE1/2-K). Any *in vitro* signal should then be interpreted using exposure-based risk assessment to determine whether a clinically meaningful DDI study is warranted.

*In vitro* data have evaluated the influence of PF on CYP3A4 and CYP2D6 at the levels of expression and enzymatic activity, indicating that PF can modulate these CYPs to varying degrees, although the magnitude appears less pronounced than that observed for related plant metabolites such as albiflorin (Gao et al., 2015). These findings warrant a cautious interpretation of “favorable safety” in the context PF, the principal bioactive component of *Paeonia* speciesof polypharmacy, and they support systematic DDI screening rather than assuming negligible interaction risk. In addition, existing pharmacokinetic analyses have discussed that low intestinal permeability and limited exposure of PF may relate not only to physicochemical features but also potentially to efflux transport processes (e.g., P-gp–mediated efflux), which—if confirmed—could further influence interaction risk with transporter inhibitors/inducers (Zhou et al., 2020).

Relevance to DPN polypharmacy. In DPN, the practical concern is interaction risk under polypharmacy (Satapathy et al., 2025). From a PK-based perspective, priority scenarios include co-medications that are CYP3A substrates or transporter substrates with narrow therapeutic windows, as exposure changes may translate into clinically meaningful toxicity or loss of efficacy (Chu et al., 2009). This is particularly relevant for commonly co-administered drug classes in DPN management, including antiplatelet/anticoagulant therapies, antihypertensives, and lipid-lowering agents, as well as symptomatic treatments for neuropathic pain (Tesfaye and Kempler, 2023). At present, available evidence is insufficient to conclude that PF has negligible DDI liability; therefore, safety claims should be framed as provisional pending systematic CYP/transporter interaction profiling at clinically relevant exposures.

Overall, available toxicological evidence supports a favorable baseline safety profile for PF in preclinical models, but a clinically meaningful translational safety narrative for DPN should explicitly incorporate polypharmacy-linked DDI liabilities, exposure–safety relationships, and special-population considerations.

### 6.3 Pharmacokinetic challenges and development implications

Clinical translation of PF is constrained by biopharmaceutic and pharmacokinetic liabilities. PF exhibits poor absorption, with very low reported oral bioavailability (∼3–4%) after oral administration (Liu et al., 2005). Consistent with this, intestinal permeability is low *in vitro*: studies using Caco-2 monolayers indicate that the apparent permeability coefficient of PF is far below commonly used high-permeability thresholds (Cheng et al., 2016). Together, these observations suggest that limited intestinal absorption and substantial presystemic loss are likely major contributors to low systemic exposure; however, quantitative physicochemical descriptors (e.g., logP/logD, polar surface area, hydrogen-bonding capacity) and gastrointestinal stability data are not consistently reported across studies. This limits rigorous biopharmaceutic classification (e.g., BCS/BDDCS) and prevents a definitive identification of the absorption rate-limiting step. Based on the combination of low oral bioavailability and very low Caco-2 permeability (Cheng et al., 2016), the current evidence most consistently supports permeability-limited absorption with substantial presystemic conversion/first-pass loss as the dominant contributors to low exposure. Accordingly, oral development would likely require strategies that improve effective permeability and/or reduce presystemic loss, whereas alternative routes may bypass some gastrointestinal barriers but still require demonstration of adequate exposure at peripheral nerve/DRG targets.

**FIGURE 3:**
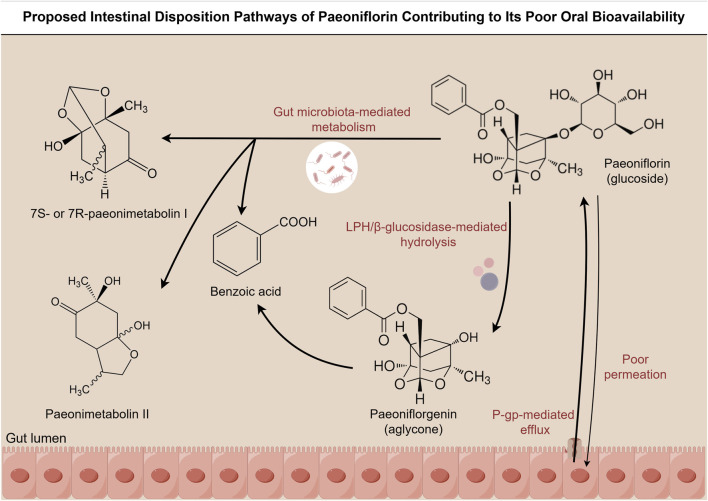
Proposed Intestinal Disposition Pathways of Paeoniflorin Contributing to Its Poor Oral Bioavailability. Schematic illustration of the proposed metabolic pathways of PF in the intestinal lumen. After oral administration, PF undergoes hydrolysis mediated by gut microbiota enzymes such as LPH and β-glucosidase, resulting in the deglycosylated form, paeoniflorigenin. Paeoniflorigenin may undergo additional metabolism, generating metabolites like benzoic acid. Furthermore, gut microbiota metabolism of PF leads to the formation of 7S- or 7R-paeonimetabolin I and paeonimetabolin II, with benzoic acid also produced. The figure also includes the contributions of poor permeability and P-glycoprotein (P-gp)-mediated efflux, which further reduce systemic exposure to PF.

As summarized schematically in Figure 3. PF undergoes extensive biotransformation, primarily through rapid hydrolysis by gut microbiota-associated carboxylesterases, generating smaller metabolites (e.g., benzoic acid) and aglycone species, which contribute to its low systemic bioavailability (Liu et al., 2006; Yu et al., 2019).

Given PF’s low oral bioavailability and extensive presystemic conversion, we hypothesize that therapeutic effects observed after oral dosing may be mediated by both parent PF and/or its metabolites, rather than by PF alone. At present, direct evidence linking specific molecular species (parent versus defined metabolites) to DPN-relevant pharmacodynamic outcomes is limited, and exposure–activity relationships across molecular species in DPN-relevant models remain to be established. This uncertainty is treated as a major translational gap and is highlighted in the evidence appraisal tables.

Inter-individual variability in gut microbiome composition can influence absorption and metabolic fate for many drugs (Jourova et al., 2016; Zhou et al., 2023). However, PF-specific evidence linking the Bacteroidetes/Firmicutes ratio to PF bioavailability remains limited, suggesting that microbiome-informed dosing is still a hypothesis requiring further validation (Walsh et al., 2018).

These properties collectively imply that (i) route selection and formulation design must explicitly address absorption and presystemic loss, (ii) metabolism-informed pharmacology is required to clarify whether efficacy in DPN is driven by PF itself or by downstream molecular species, and (iii) translational readiness should be framed conservatively until DPN-relevant exposure–response relationships (including peripheral nerve and/or DRG exposure, where feasible) are established. At present, pharmacokinetic parameters of PF in DPN-relevant models are incompletely characterized. Specifically, key PK metrics (Cmax, AUC, clearance, volume of distribution, and half-life) and, critically, peripheral nerve and/or DRG concentrations are rarely reported alongside pharmacodynamic endpoints. This lack of DPN-contextualized PK data constitutes a major translational gap, limiting rational dose selection and interpretation of efficacy, and should be addressed in future PK–PD studies.

### 6.4 Strategies to address exposure limitations and strengthen translational feasibility

#### 6.4.1 Formulation and delivery strategies: current evidence gaps and priorities

Given PF’s low oral bioavailability and low permeability, formulation and delivery optimization remains a plausible development direction. However, it is essential to distinguish PF-specific evidence from platform-level delivery concepts. At present, direct evidence demonstrating that specific delivery systems increase PF exposure at the intended peripheral targets (peripheral nerve and/or dorsal root ganglia) and translate into disease-relevant DPN outcomes remains limited (Liu et al., 2018). Therefore, future studies should prioritize PF-specific validation in DPN-relevant models, including: (i) quantifying systemic exposure (Cmax, AUC) and, where possible, peripheral nerve/DRG concentrations; (ii) linking exposure to meaningful endpoints beyond pain behavior (e.g., nerve conduction, intraepidermal nerve fiber density, myelin/axon integrity); and (iii) benchmarking against appropriate controls, including vehicle and positive comparators.

For a primarily peripheral indication such as DPN, delivery strategies should also incorporate a benefit–risk rationale regarding central exposure. Enhanced BBB penetration is not necessarily advantageous when the primary therapeutic target is peripheral nerve tissue, and increased CNS exposure could plausibly increase the risk of CNS-related adverse effects (Liu and Sahi, 2025). Accordingly, if a formulation is designed to increase BBB permeability, the intended therapeutic objective (central pain processing vs. peripheral nerve repair), expected benefit, and safety monitoring plan should be explicitly justified.

#### 6.4.2 Structural optimization strategies: Derivatives and prodrugs

In addition to formulation-based approaches, structural optimization may provide alternative or complementary strategies to improve systemic exposure (Xiao et al., 2026). For compounds with poor permeability and/or extensive presystemic metabolism, prodrug design (e.g., transient masking of polar functionalities to enhance membrane permeability, followed by enzymatic conversion to the active entity) can improve absorption and oral exposure, while potentially enabling tissue-selective activation. Likewise, rational derivative design may target improved metabolic stability, reduced efflux susceptibility, or enhanced distribution to peripheral neural tissues (Liu et al., 2018; Zhang and Tang, 2018). Importantly, such strategies require careful confirmation of the active molecular entity (parent vs. metabolite) and should be coupled to pharmacokinetic–pharmacodynamic (PK–PD) evaluation in DPN-relevant models to ensure that increases in exposure translate into meaningful disease-modifying endpoints rather than nonspecific effects.

## 7 Multitarget mechanisms of PF in the treatment of DPN

**FIGURE 4:**
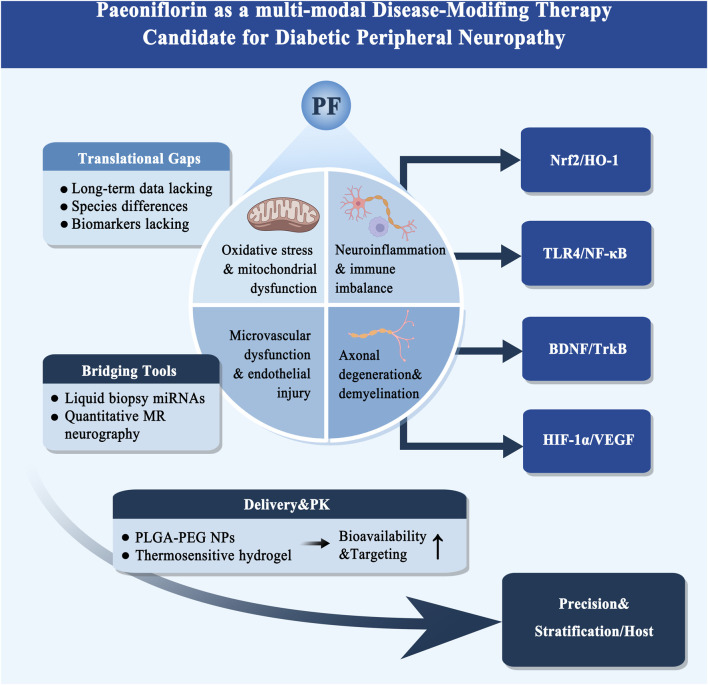
Paeoniflorin as a Multimodal Disease-Modifying Therapy Candidate for Diabetic Peripheral Neuropathy. The diagram summarizes a precision-to-translation framework for PF in DPN. mitochondrial dysfunction, (ii) neuroinflammation and immune imbalance, (iii) microvascular dysfunction and endothelial injury, and (iv) axonal degeneration and demyelination. At the top, current symptomatic drugs (pregabalin, duloxetine) lower pain scores but do not reverse structural pathology. On the right, PF engages four disease-modifying axes: activation of Nrf2/HO-1 (antioxidant defense), inhibition of TLR4/NF-κB (re-balances the neuroimmune milieu), upregulation of BDNF/TrkB (axonal regeneration and myelin repair), and modulation of HIF-1α/VEGF (endothelial protection and microcirculation support). The left and bottom panels outline translation enablers: (1) Translational gaps (species differences, lack of robust biomarkers, limited long-term data), (2) Delivery and PK (e.g., PLGA-PEG nanoparticles, thermosensitive hydrogels) to increase bioavailability and targeting, (3) Precision and Stratification/Host (subtyping into painful small-fiber vs. motor large-fiber, plus genetics/epigenetics/microbiota), and (4) Bridging tools (liquid-biopsy miRNAs and quantitative MR neurography) used for enrollment, pharmacodynamic monitoring, and longitudinal follow-up. Arrows denote activation/flow; red bar denotes inhibition; green modules indicate PF-mediated protective or reparative actions.

PF, the principal plant-derived metabolite Paeonia species, exerts multitarget neuroprotective effects against DPN. Acting through a coordinated network of antioxidative, anti-inflammatory, neurotrophic, and vasoprotective pathways, PF activates the Nrf2/HO-1 axis to alleviate oxidative stress, inhibits the TLR4/NF-κB cascade to rebalance neuroimmune responses, enhances BDNF/TrkB-mediated axonal regeneration and synaptic repair, and modulates HIF-1α/VEGF and eNOS signaling to preserve microvascular integrity. These integrated actions mitigate mitochondrial dysfunction, inflammation, demyelination, and ischemia, positioning PF as a promising multitarget therapeutic candidate for DPN (Figure 4). The methodological strengths and limitations of studies supporting this pathway are summarized in Supplementary Table S1, with direct DPN efficacy studies distinguished from non-DPN mechanistic evidence.

### 7.1 Antioxidative stress and mitochondrial protection

PF exerts antioxidative effects primarily through the activation of the nuclear factor erythroid 2–related factor 2 (Nrf2)/heme oxygenase-1 (HO-1) signaling pathway, a mechanism demonstrated in multiple models of diabetic complications. Nrf2 translocates to the nucleus and induces HO-1 expression, thereby scavenging reactive oxygen species (ROS) and mitigating oxidative injury to Schwann cells. Studies in high-glucose-induced Schwann cell injury models have shown that PF significantly upregulates Nrf2 and HO-1 expression, reduces ROS production, preserves mitochondrial integrity, and alleviates neuronal damage. Moreover, activation of this pathway interacts with downstream glutathione metabolism and anti-apoptotic mechanisms, forming a key component of PF’s protective effects against DPN (Yang et al., 2016; Tavakkoli et al., 2019).

Protection of mitochondrial function represents another crucial mechanism by which PF mitigates the pathological progression of DPN. Evidence indicates that PF alleviates mitochondrial dysfunction under hyperglycemic conditions—specifically the loss of mitochondrial membrane potential and impaired ATP synthesis—thereby maintaining the energy homeostasis of neurons and Schwann cells (Akki et al., 2022). This protective effect is likely mediated through the upregulation of mitochondrial dynamics–related proteins such as MFN2 and OPA1, along with the suppression of mitochondrial ROS production. Although direct studies investigating PF’s restoration of mitochondrial oxidative phosphorylation in DPN models remain limited, experimental findings from other neurodegenerative disease models—such as Parkinson’s disease and cerebral ischemia–reperfusion injury—have demonstrated that PF upregulates mitochondrial biogenesis factors, including PGC-1α and Nrf1, thereby promoting ATP synthesis and mitochondrial regeneration (Zhang L. et al., 2024). These results provide valuable mechanistic insights that may be extrapolated to DPN pathology.

PF enhances antioxidative defense by modulating the glutathione (GSH) metabolism system, notably increasing the activity of glutathione peroxidase (GPX) to eliminate ROS products such as hydrogen peroxide (H_2_O_2_). In diabetic nephropathy models, PF markedly upregulates GPX activity, an effect shown to act synergistically with the Nrf2–HO-1 pathway (Zhang X. E. et al., 2023). This mechanism is potentially applicable to the high-glucose–induced neuroinflammatory context of DPN, although direct evidence specific to DPN models remains limited.

In summary, PF effectively mitigates hyperglycemia-induced oxidative stress through three complementary mechanisms: activation of the Nrf2/HO-1 antioxidative signaling axis, stabilization of mitochondrial function, and reconstruction of the glutathione redox system. This continuous antioxidative cascade—“signal activation–energy stabilization–redox system reconstruction”—helps comprehensively suppress early-stage DPN pathologies such as neural lipid peroxidation and oxidative DNA damage, providing multi-level protection for axonal survival and functional recovery. Although the key nodes of these pathways have been validated in multiple *in vitro* and *in vivo* studies, their specific roles in DPN still require clarification through tissue-specific localization and time-course analyses. Such investigations will be essential to determine the breadth and selectivity of paeoniflorin’s antioxidative effects and to establish a theoretical foundation for its clinical translation in diabetic neuropathy therapy.

### 7.2 Anti-inflammatory and immunomodulatory effects

PF exhibits pronounced anti-inflammatory and immunomodulatory activity in the treatment of DPN, primarily by inhibiting the TLR4/NF-κB inflammatory signaling axis and modulating macrophage polarization balance (Shao et al., 2019; Hong et al., 2022; Liu Y. et al., 2025; Ou et al., 2025).

In streptozotocin (STZ)-induced diabetic nephropathy (DN) models, PF significantly downregulates the expression of TLR4, MyD88, and NF-κB p65 in renal tissues, while reducing M1 macrophage-associated markers such as iNOS, TNF-α, and IL-1β(Shao et al., 2019). Although this study focused on the kidneys, TLR4-mediated inflammatory mechanisms are also widely implicated in DPN, suggesting that PF may suppress immune activation in the peripheral nervous system through a similar pathway. Moreover, in high-glucose-stimulated RAW264.7 macrophages, PF effectively inhibits the activation of downstream TLR4 molecules, including p-IRAK1, TRIF, and NF-κB p-p65, thereby reducing the secretion of proinflammatory cytokines (Zhang et al., 2017).

At the same time, previous reviews (Ou et al., 2025) have reported that PF can block the TLR4–MyD88–NF-κB signaling cascade, alleviating high-glucose-induced Schwann cell apoptosis, further supporting its neuroprotective role in peripheral nerves. In diabetic rat sciatic nerves, PF (30 mg/kg) markedly decreases infiltration of CD68^+^ macrophages and downregulates M1 phenotype markers such as iNOS and IL-6, while upregulating M2 markers including Arg-1 and IL-10. This effect is strongly associated with inhibition of the TLR4/NF-κB pathway (Ou et al., 2025).

Hong et al. (2022) further summarized PF’s regulatory effects on spinal microglial polarization, showing that PF suppresses TLR4-mediated NF-κB activation and reduces the proportion of M1-type microglia (a mechanism similar to that of platelet-rich plasma therapy in promoting M2 polarization and reducing inflammation (Dou and An, 2025)), thereby alleviating diabetes-related neuroinflammatory pain. Beyond classical inflammatory signaling, PF may also exert synergistic effects through activation of the Nrf2/ARE antioxidant pathway or the PPARγ signaling axis, further modulating macrophage functional states and attenuating chronic inflammation in DPN (Ou et al., 2025).

Taken together, PF primarily suppresses M1 macrophage polarization through a TLR4/NF-κB–dependent mechanism and may act cooperatively through auxiliary pathways such as Nrf2/ARE. Future investigations should incorporate TLR4 knockout models and molecular docking analyses to determine whether PF directly interferes with ligand–receptor interactions between HMGB1 and TLR4.

### 7.3 Neuroregeneration and synaptic functional reconstruction

PF exerts neuroprotective effects in DPN not only through its anti-inflammatory and antioxidative activities but also by promoting neural structural repair and synaptic functional recovery via multitarget regulatory mechanisms. Studies have shown that PF enhances axonal growth, activates neurotrophic signaling pathways, and improves synaptic plasticity, thereby synergistically facilitating the regeneration and functional restoration of damaged nerves (Liu et al., 2019; Hong et al. 2022; Alhamdi et al., 2025; Choi et al., 2025).

In terms of neurotrophic regulation, PF markedly upregulates the expression of brain-derived neurotrophic factor (BDNF) and its high-affinity receptor TrkB, thereby activating the BDNF/TrkB signaling pathway and enhancing the phosphorylation of cAMP response element-binding protein (CREB). This activation triggers a cascade of downstream neuroplasticity-related processes (Choi et al., 2025). Previous studies have confirmed that PF-containing formulations can stimulate the BDNF/TrkB/PI3K signaling axis, significantly increasing the expression of synaptic proteins such as PSD-95 and synaptophysin, thus promoting axonal regeneration, synaptic connectivity, and functional recovery (Meng et al., 2023).

Beyond BDNF, PF also regulates another critical neurotrophic factor—neurotrophin-3 (NT-3)—and enhances its binding affinity with the TrkC receptor, thereby activating pathways involved in Schwann cell differentiation and myelination. In related neurorepair studies, Trueman et al. (Alhamdi et al., 2025) reported that PF treatment significantly activated the NT-3/TrkC axis, induced Schwann cells to synthesize the key myelin protein P0, and effectively mitigated demyelination observed in diabetic and nerve injury models, promoting peripheral nerve remyelination. This mechanism may have clinical significance in improving reduced nerve conduction velocity and sensory dysfunction commonly seen in DPN.

Collectively, PF exerts a multitarget neuroprotective effect by coordinating multiple signaling pathways that regulate neurotrophic factor expression, axonal regeneration, Schwann cell remyelination, and synaptic remodeling. These integrated actions constitute a key mechanism underlying its therapeutic potential in DPN.

### 7.4 Microcirculatory improvement and vascular protection

As the principal plant-derived metabolite of *Paeonia* species, PF has demonstrated a wide spectrum of pharmacological activities related to vascular protection and microcirculatory enhancement in recent years. Multiple studies have shown that PF simultaneously modulates inflammatory and oxidative stress pathways to regulate endothelial function via multiple signaling networks. Liu et al. reported that PF alleviates endothelial inflammation by inhibiting the NF-κB signaling pathway while activating the Nrf2/HO-1 axis to reduce ROS levels. This dual modulation indirectly enhances endothelial nitric oxide synthase (eNOS) activity, promoting nitric oxide (NO) production and improving vascular relaxation (Liu Z. et al., 2025). Moreover, Zhang et al. found that PF regulates the TGF-β1/Smad and MAPK pathways, thereby reducing endothelial injury and collagen deposition—suggesting a multitarget protective role in chronic inflammatory vasculopathies (Zhang et al., 2025).

In microvascular disease models, PF exhibits strong anti-leakage and anti-angiogenic effects. In diabetic retinopathy models, PF inhibits ROS generation and blocks NF-κB–mediated HIF-1α nuclear translocation and VEGF-A transcriptional activation, leading to significant suppression of VEGF-A expression and attenuation of pathological retinal neovascularization (Song et al., 2017). Additionally, PF suppresses the HIF-1α/p-STAT3 axis, further inhibiting abnormal angiogenesis. By restoring mitochondrial membrane potential and ATP content, PF stabilizes endothelial junctions and improves the integrity of the blood–retina barrier (Kong et al., 2022).

Systemically, PF also enhances hemodynamics and microcirculatory perfusion. Li et al. reported that PF significantly improved tail microvascular perfusion in spontaneously hypertensive rats (SHR), an effect closely associated with the upregulation of eNOS expression, suggesting that PF may relieve microcirculatory disturbances by improving hemorheological properties (Li et al., 2018). Furthermore, PF promotes the release of endothelial-derived hyperpolarizing factors (EDHF), strengthens small-vessel relaxation responses, and restores neurotrophic vascular function, providing notable protection to ischemic neural tissue (Bartáková and Nováková, 2021).

In summary, PF acts through multiple key signaling pathways—including NF-κB, Nrf2/HO-1, HIF-1α/VEGF-A, TGF-β/Smad, and eNOS/EDHF—to exert anti-inflammatory, antioxidative, vasodilatory, and anti-angiogenic effects. Through these coordinated actions, PF systematically improves endothelial function and microcirculatory status, providing a solid theoretical foundation for its potential application in diabetic complications and hypertensive vascular disorders.

## 8 Synergistic strategies: combination therapy approaches

PF, as a key bioactive monoterpene glycoside, serves as a active metabolite in several botanical drug preparations used in traditional Chinese medicine (TCM) formulations that demonstrate multitarget and synergistic therapeutic potential in DPN and peripheral nerve injury repair.

Compound Danshen Dripping Pill (CDDP) has been reported in multiple randomized controlled trials (RCTs) and systematic reviews to improve microcirculatory parameters in patients with type 2 diabetes and exert positive effects on early peripheral neuropathy, as evidenced by improvements in subjective symptoms and partial electrophysiological indices (Huang et al., 2021).

Xuebijing Injection, a compound TCM injection, has shown the ability to downregulate IL-6 and TNF-α while enhancing antioxidant enzyme activities such as superoxide dismutase (SOD) and glutathione peroxidase (GSH-Px) across various inflammatory models. These combined anti-inflammatory and antioxidant effects provide a mechanistic rationale for mitigating diabetes-related tissue injury (Kang et al., 2023).

Shaoyao-Gancao Decoction, another PF-containing formula, has demonstrated analgesic and neuroprotective effects in chemotherapy-induced and inflammatory pain models by inhibiting TRPV1 expression and associated inflammatory signaling, thereby reducing neuropathic pain. This offers preclinical evidence supporting its potential utility in managing peripheral neuropathic pain syndromes (Chen et al., 2022).

Collectively, PF-centered compound formulations exhibit synergistic effects through multiple complementary mechanisms, including enhancement of microcirculation, upregulation of neurotrophic factors, suppression of inflammatory pathways, and reinforcement of antioxidative defenses. These combined actions contribute to systemic neurorepair and improvement in functional outcomes. Thus, PF-based combination therapies hold promise as adjuvant or synergistic strategies for DPN management. Nevertheless, well-designed randomized controlled clinical trials and mechanistic studies are warranted to delineate their efficacy spectrum, optimize formulation ratios, and confirm safety for translational application.

## 9 Preclinical evidence for PF mechanisms: Strengths and heterogeneity

Although numerous preclinical studies consistently report that PF mitigates oxidative stress through activation of the Nrf2/HO-1 pathway and remodels the immune microenvironment via inhibition of the TLR4/NF-κB axis (Shao et al., 2019; Choi et al., 2025), existing evidence demonstrates substantial heterogeneity that may overstate its translational potential.

First, the variability among animal models significantly limits the generalizability of findings. Streptozotocin (STZ)-induced models primarily simulate acute hyperglycemic injury, whereas genetic or diet-induced type 2 diabetes models better reflect chronic metabolic overload. These two categories differ fundamentally in neuropathic phenotype and regenerative potential. Consequently, interventions validated in only one model type may yield biased assessments of clinical feasibility (Sullivan et al., 2007). Such inter-model discrepancies amplify result inconsistency and weaken translational reliability.

Second, methodological shortcomings are prevalent across studies, including inadequate reporting of blinding and randomization procedures and the use of small sample sizes (typically *n* = 6–12). Methodological meta-research indicates that such deficiencies systematically inflate positive effect estimates in small-sample experiments (Hooijmans et al., 2014). Hence, isolated small-scale positive findings should not be interpreted as robust therapeutic signals.

Third, dose–exposure discrepancies hinder translational extrapolation. Several *in vitro* and *in vivo* studies have used PF concentrations or doses (10–100 mg kg^-1^ *in vivo* or >10 μM *in vitro*) far exceeding exposure levels achievable in humans (Li et al., 2017; Ou et al., 2025). Moreover, interspecies differences in metabolism and bioavailability between rodents and humans introduce additional bias when extrapolating pharmacokinetic outcomes (Ou et al., 2025).

Lastly, the reporting of safety data and negative results remains insufficient. Some studies have noted fluctuations in hepatic enzyme levels or signs of immunosuppression at high PF doses; however, such adverse events and interspecies differences are frequently overlooked or not systematically disclosed (Ou et al., 2025). Consequently, to accurately determine the true therapeutic efficacy of PF in diabetic complications, including DPN, a more rigorous and standardized preclinical evidence framework is urgently needed.

Specifically, (1) representative and multimodal animal models—such as the combination of a high-fat diet with low-dose streptozotocin (STZ)—should be employed to better replicate the chronic metabolic disturbances characteristic of type 2 diabetes. (2) Experimental designs should strictly incorporate randomization and blinding procedures and adhere to established methodological standards such as the SYRCLE guidelines to ensure transparency and reproducibility. (3) Comprehensive toxicological and pharmacokinetic evaluations should be integrated to align experimental exposures with clinically achievable levels, thereby enhancing translational relevance. (4) Systematic preclinical reviews and meta-analyses should be conducted to quantify sources of heterogeneity and include negative or neutral findings, reducing publication bias and overestimation of efficacy.

Only when such rigorous methodological and evidentiary standards are satisfied can the progression of PF into early-phase clinical trials be considered both scientifically sound and ethically justified.

## 10 Challenges in clinical translation and perspectives on personalized medicine

### 10.1 Bottlenecks in translational medicine

PF has shown favorable effects on peripheral nerve structure and function in various animal models. However, translating these findings from animals to humans faces several key limitations.

First, inter-species differences in the blood-nerve and blood-ganglion barriers impact local drug exposure and tissue area under the curve (AUC). The expression of tight junction proteins (such as claudin-5) in the blood-nerve barrier varies significantly between species and between different regions within a species, thus affecting the permeability and tissue accumulation of both small molecules and nanoparticle carriers (Martignoni et al., 2006; Lux et al., 2019).

Second, species differences in drug-metabolizing enzymes make it challenging to extrapolate pharmacokinetics and pharmacodynamics (PK/PD) data from rodents to humans. The CYP family (including the CYP3A subtypes) exhibits significant interspecies variability in catalytic capacity and isoform phenotypes, altering PF’s clearance and first-pass effect, which impacts both systemic and local exposure. Studies on drug–drug interactions, together with *in situ*/*ex vivo* intestinal studies, suggest that P-glycoprotein (P-gp) is involved in the intestinal disposition of PF *in vivo*, whereas the involvement of CYP3A4 has been inferred mainly from *in vitro* human-derived systems and rat *in vivo* interaction studies, highlighting the need for specific evaluation of metabolic clearance and transporter interactions in humans (Liu et al., 2006; Gao et al., 2015; Chen et al., 2021).

Third, the limited comparability of pain and analgesia endpoints across species poses another challenge. Common animal behavioral endpoints (e.g., mechanical/thermal thresholds, paw withdrawal reflex) are objective and convenient, but these do not directly correlate with human subjective pain assessments (e.g., VAS/NPQ). As a result, animal data has limited predictive power for clinical analgesic efficacy, leading to systematic bias in efficacy forecasting. Reviews have pointed out that animal pain models differ fundamentally from human pain experiences, both biologically and psychosocially, and must be interpreted with caution (Berge, 2011).

In addition to these interspecies challenges, current clinical research suffers from an inadequate biomarker system for efficacy or biological response monitoring. Several commonly used biomarkers have significant limitations: inflammatory mediators (such as TNF-α) have short half-lives and fluctuate in circulation, making single-point measurements insufficient to reflect dynamic inflammatory states; cytokines like IL-6 also show short half-lives, requiring highly standardized sampling times and conditions (Morozova et al., 2024).

Oxidative stress markers (e.g., urinary 8-OHdG) and regenerative endpoints face issues with variability and insufficient specificity. The levels of urinary 8-OHdG fluctuate significantly within a day and are influenced by diet and lifestyle, making single measurements inadequate. Epidermal nerve fiber density (IENFD), a commonly used structural endpoint, also exhibits considerable variation between different anatomical regions and individuals, with sampling and counting techniques having a marked impact on results (Li et al., 2021).

To overcome these bottlenecks, a multimodal evaluation system is recommended, incorporating tissue/fluids biomarkers, quantitative imaging, and minimally invasive/dynamic sampling techniques. Exosome/circulating miRNAs (such as the miR-30c series, which has been shown in animal studies to correlate with Schwann cell remyelination and nerve repair) are promising candidates. However, there is a lack of strict clinical validation linking PF treatment responses to specific exosome miRNAs (e.g., miR-30c). Therefore, these miRNAs should be validated as candidate biomarkers in preclinical and early clinical studies (Tramullas et al., 2018; Fan et al., 2020).

Regarding imaging, 3.0 T MR neuroimaging (MRN) and its quantitative indices (such as T2 relaxation time or DTI/FA metrics) can non-invasively reflect edema, nerve fiber integrity, and microstructural changes in peripheral nerves. Current studies have established reference values for MRN and cross-scanner reproducibility assessments, providing a feasible pathway for incorporating imaging endpoints into PF clinical trials. However, further validation of sensitivity and specificity for DPN and PF intervention is required (Sollmann et al., 2020; Preisner et al., 2022).

Finally, minimally invasive dynamic monitoring techniques such as interstitial/subcutaneous microdialysis can be used for real-time or semi-continuous measurements of local cytokines (e.g., IL-1β, IL-6) and metabolites. This helps clarify the immediate drug effects and inflammatory dynamics, guiding optimization of dosage and administration strategies. Existing brain/muscle/skin microdialysis methods have proven capable of recovering and quantifying cytokines, but the feasibility and standardization of peripheral nerve microdialysis in clinical settings still require methodological refinement (Winter et al., 2002; Hersini et al., 2014).

## 11 Conclusion and prioritized next steps

PF exhibits multi-pathway biological activity that overlaps with key pathological processes implicated in DPN, including oxidative stress, neuroinflammation, neurotrophic signaling, and microvascular dysfunction. Nevertheless, the current body of evidence remains predominantly preclinical, heterogeneous in methodological quality, and limited in DPN-contextualized pharmacokinetic characterization. Accordingly, translational conclusions should be interpreted with caution.

To strengthen the evidence base and improve translational interpretability, several priorities merit attention. First, the pharmacologically active molecular entity should be clarified, particularly in light of extensive presystemic biotransformation, through metabolite-resolved exposure–activity analyses in DPN-relevant models. Second, pharmacokinetic–pharmacodynamic integration should be improved by reporting systemic exposure parameters alongside functional and structural neuropathy endpoints, including, where feasible, peripheral nerve or dorsal root ganglion concentrations. Third, future studies should enhance methodological rigor through appropriate randomization, blinding, sample size justification, and use of disease models reflecting chronic type 2 diabetes–associated neuropathy. Fourth, formulation strategies should be supported by PF-specific validation in DPN models rather than extrapolated from platform-level concepts, and disease-modifying endpoints beyond pain behavior should be prioritized. Finally, given the polypharmacy context of DPN, systematic assessment of CYP- and transporter-mediated interaction potential is warranted to support a clinically realistic safety narrative.

In summary, PF represents a biologically plausible candidate deserving further investigation; however, its progression toward clinical evaluation requires more rigorous mechanistic clarification, exposure-driven validation, and safety profiling aligned with established pharmacological standards.

## 12 Discussion

PF shows biologically plausible, multi-pathway activity that overlaps with major pathological domains implicated in DPN. Across preclinical studies, PF has been reported to activate antioxidative and cytoprotective signaling (e.g., Nrf2/HO-1) and to attenuate inflammatory cascades (e.g., TLR4/NF-κB), providing a mechanistic rationale for considering PF as a multi-target candidate within a disease-modifying framework (Yang et al., 2016; Shao et al., 2019). Nevertheless, the current literature remains heterogeneous in models, endpoints, and reporting quality, and therefore should be interpreted primarily as mechanistic and hypothesis-generating rather than development-confirmatory evidence.

A central translational constraint is PF’s biopharmaceutic and pharmacokinetic profile. Low oral bioavailability, limited intestinal permeability, and extensive presystemic processing may substantially limit systemic exposure and complicate dose–response interpretation across studies (Liu et al., 2005; Liu et al., 2006). Moreover, potential efflux processes (e.g., P-gp–related) and metabolic conversion raise uncertainty regarding the active molecular entity after oral dosing, which weakens causal linkage between administered PF and observed pharmacodynamic effects unless exposure is quantified in a DPN-relevant context. Consequently, mechanistic claims are most convincing when supported by appropriate dose/concentration reporting, adequate controls, and endpoints that map onto neuropathy-relevant structural and functional changes.

Formulation and delivery approaches remain conceptually attractive to address exposure limitations, but a key interpretive issue is the frequent reliance on platform-level delivery concepts rather than PF-specific validation in DPN models. For a peripheral indication such as DPN, delivery strategies should be assessed against a clear target rationale (peripheral nerve/DRG versus central pain processing) and should demonstrate PF-specific improvements in exposure aligned with disease-relevant outcomes beyond pain behavior (e.g., nerve conduction, intraepidermal nerve fiber density, and myelin/axon integrity). Without such PF-specific PK–PD and endpoint linkage, delivery discussions risk overstating translational readiness.

Another major limitation is endpoint and biomarker heterogeneity. Commonly used measures (e.g., nerve conduction velocity, IENFD, and circulating inflammatory markers) each carry known variability and interpretive constraints, and emerging tools such as quantitative MR neuroimaging or minimally invasive sampling techniques have not yet been consistently integrated with pharmacokinetics, target engagement, and functional outcomes into a unified evidentiary chain (Winter et al., 2002; Kelley and Hackshaw, 2021; Preisner et al., 2022). This fragmentation contributes to the broader, well-recognized challenge of high attrition from preclinical signals to clinical benefit, particularly in chronic, heterogeneous conditions where species differences, comorbidities, and polypharmacy complicate external validity (Ineichen et al., 2024).

Overall, the key challenge is not the absence of plausible mechanisms but the need for more rigorous, exposure-informed and endpoint-aligned evidence that supports reproducible interpretation across studies. A more credible translational narrative for PF in DPN will therefore depend on (i) clarifying the active molecular entity and DPN-contextualized exposure, (ii) improving methodological rigor and model relevance, and (iii) linking exposure to disease-modifying structural and functional outcomes using clinically meaningful measures.

## Glossary

5-HT: 5-Hydroxytryptamine (Serotonin)
8-OHdG: 8-Hydroxy-2′-deoxyguanosine
AGE: Advanced Glycation End products
ARE: Antioxidant Response Element
ASC: Apoptosis-associated Speck-like protein containing a CARD
ASK1: Apoptosis Signal-regulating Kinase 1
BDNF: Brain-Derived Neurotrophic Factor
CAP: Capsaicin (in context of TRPV1 currents)
CCL2: C-C Motif Chemokine Ligand 2
CCR2: C-C Chemokine Receptor Type 2
CD68: Cluster of Differentiation 68
CMAP: Compound Muscle Action Potential
CRMP2: Collapsin Response Mediator Protein 2
CS/β-GP: Chitosan/β-Glycerophosphate
CX3CL1: Chemokine (C-X3-C motif) ligand 1
CX3CR1: Chemokine (C-X3-C motif) receptor 1
CYP2D6: Cytochrome P450 2D6
CYP3A4: Cytochrome P450 3A4
DMTs: Disease-Modifying Therapies
DPN: Diabetic Peripheral Neuropathy
DRG: Dorsal Root Ganglion
ErbB2: Erb-B2 Receptor Tyrosine Kinase 2
FREMS: Frequency Rhythmic Electrical Modulated System
GDNF: Glial cell line-Derived Neurotrophic Factor
GFAP: Glial Fibrillary Acidic Protein
HbA1c: Hemoglobin A1c
HDAC: Histone Deacetylase
HDAC1: Histone Deacetylase 1
HDAC4: Histone Deacetylase 4
HDAC6: Histone Deacetylase 6
HF: High Frequency (in HRV context)
HIF-1α: Hypoxia-Inducible Factor 1-alpha
HO-1: Heme Oxygenase-1
HRV: Heart Rate Variability
HVA: High-Voltage-Activated (calcium channels)
ICAM-1: Intercellular Adhesion Molecule 1
IDF: International Diabetes Federation
IENFD: Intraepidermal Nerve Fiber Density
IκB: Inhibitor of kappa B
IKKβ: IκB kinase beta
IL-1β: Interleukin-1 beta
IL-18: Interleukin-18
IL-6: Interleukin-6
IRAK1: Interleukin-1 Receptor-Associated Kinase 1
JNK: c-Jun N-terminal kinase
LD50: Median Lethal Dose
LPH: Lactase phlorizin hydrolase
MBP: Myelin Basic Protein
MCP-1: Monocyte Chemoattractant Protein-1
miR-128: microRNA-128
miR-146a: microRNA-146a
miR-30: microRNA-30
miRNAs: microRNAs
MMP-9: Matrix Metalloproteinase-9
MnSOD: Manganese Superoxide Dismutase
mtDNA: Mitochondrial DNA
Nav1.7: Voltage-gated sodium channel 1.7
Nav1.8: Voltage-gated sodium channel 1.8
NE: Norepinephrine
NF-κB: Nuclear Factor kappa-B
NGF: Nerve Growth Factor
NLRP3: NLR Family Pyrin Domain Containing 3
NOX2: NADPH Oxidase 2
Nrf2: Nuclear factor erythroid 2-related factor 2
p38MAPK: p38 Mitogen-Activated Protein Kinase
PAF: Platelet-Activating Factor
PF: Paeoniflorin
PKC: Protein Kinase C
PLGA-PEG: Poly(lactic-co-glycolic acid)-Polyethylene Glycol
QTc: Corrected QT interval
RAGE: Receptor for Advanced Glycation End products
REST: RE1-Silencing Transcription factor
RGD: Arginine-Glycine-Aspartic acid
RhoA/ROCK: Ras homolog family member A/Rho-associated protein kinase
ROS: Reactive Oxygen Species
SCN9A: Sodium Voltage-Gated Channel Alpha Subunit 9
SCS: Spinal Cord Stimulation
SDNN: Standard Deviation of Normal to Normal intervals
sFlt-1: Soluble Fms-like Tyrosine kinase 1
SGC: Satellite Glial Cell (primary context; secondary: Sodium Glyco-deoxycholate)
SNRIs: Serotonin-Norepinephrine Reuptake Inhibitors
SPC: Soybean Phosphatidylcholine
T2D: Type 2 Diabetes
TfR: Transferrin Receptor
TLR4: Toll-like receptor 4
TNF-α: Tumor Necrosis Factor-alpha
TRAF6: TNF Receptor-Associated Factor 6
TREM2: Triggering Receptor Expressed on Myeloid cells 2
TrkA: Tropomyosin receptor kinase A
TrkB: Tropomyosin receptor kinase B
TRPV1: Transient Receptor Potential Vanilloid 1
TSA: Trichostatin A
UC: Ulcerative Colitis
VEGF: Vascular Endothelial Growth Factor
VEGF165: Vascular Endothelial Growth Factor 165
VEGFR-2: Vascular Endothelial Growth Factor Receptor 2
VGCC: Voltage-Gated Calcium Channels
ZO-1: Zonula Occludens-1
ΔΨm: Mitochondrial membrane potential
P-gp: P-glycoprotein
**pO**_**2**_: Partial pressure of oxygen

## References

[B1] Abd Razak N. H. Idris J. Hassan N. H. Zaini F. Muhamad N. Daud M. F. (2024). Unveiling the role of schwann cell plasticity in the pathogenesis of diabetic peripheral neuropathy. Int. J. Mol. Sci. 25 (19), 10785. 10.3390/ijms251910785 39409114 PMC11476695

[B2] Akki R. Siracusa R. Cordaro M. Remigante A. Morabito R. Errami M. (2022). Adaptation to oxidative stress at cellular and tissue level. Arch. Physiol. Biochem. 128 (2), 521–531. 10.1080/13813455.2019.1702059 31835914

[B3] Albrecht P. J. Houk G. Ruggiero E. Dockum M. Czerwinski M. Betts J. (2021). Keratinocyte biomarkers distinguish painful diabetic peripheral neuropathy patients and correlate with topical lidocaine responsiveness. Front. Pain Res. (Lausanne) 2, 790524. 10.3389/fpain.2021.790524 35295428 PMC8915676

[B4] Alhamdi A. A. Mackie S. Trueman R. P. Rayner M. L. D. (2025). Pharmacologically targeting schwann cells to improve regeneration following nerve damage. Front. Cell Dev. Biol. 13, 1603752. 10.3389/fcell.2025.1603752 40612106 PMC12222135

[B5] Alizadeh S. D. Jahani S. Rukerd M. R. Z. Tabrizi R. Masoomi R. Banihashemian S. Z. (2024). Human studies of the efficacy and safety of stem cells in the treatment of diabetic peripheral neuropathy: a systematic review and meta-analysis. Stem Cell Res. Ther. 15 (1), 442. 10.1186/s13287-024-04033-3 39563393 PMC11577959

[B6] Alizadeh S. D. Hassan Zadeh Tabatabaei M. S. Rezaei Zadeh Rukerd M. Tabrizi R. Masoomi R. Banihashemian S. Z. (2025). The safety and efficacy of stem cell therapy for diabetic peripheral neuropathy in animal studies: a systematic review and meta-analysis. Neuroscience 566, 49–59. 10.1016/j.neuroscience.2024.12.035 39706518

[B7] Alleman C. J. Westerhout K. Y. Hensen M. Chambers C. Stoker M. Long S. (2015). Humanistic and economic burden of painful diabetic peripheral neuropathy in Europe: a review of the literature. Diabetes Res. Clin. Pract. 109 (2), 215–225. 10.1016/j.diabres.2015.04.031 26008721

[B8] Apfel S. C. (2002). “Nerve growth factor for the treatment of diabetic neuropathy: what went wrong, what went right, and what does the future hold?,” in International review of neurobiology (Academic Press), 393–413. 10.1016/s0074-7742(02)50083-0 12198818

[B9] Arimura N. Ménager C. Kawano Y. Yoshimura T. Kawabata S. Hattori A. (2005). Phosphorylation by Rho kinase regulates CRMP-2 activity in growth cones. Mol. Cell Biol. 25 (22), 9973–9984. 10.1128/mcb.25.22.9973-9984.2005 16260611 PMC1280267

[B10] Bahl S. Seto E. (2021). Regulation of histone deacetylase activities and functions by phosphorylation and its physiological relevance. Cell. Mol. Life Sci. 78 (2), 427–445. 10.1007/s00018-020-03599-4 32683534 PMC7854769

[B11] Bai F. Long Z. Yang J. Liu P. Wu Z. Chen H. (2025). Induction of mitochondrial biogenesis enhances neurogenesis and cognitive recovery following ischaemic stroke. Anesthesiol. Perioper. Sci. 3 (4), 52. 10.1007/s44254-025-00135-0

[B12] Baka P. Escolano-Lozano F. Birklein F. (2021). Systemic inflammatory biomarkers in painful diabetic neuropathy. J. Diabetes Its Complicat. 35 (10), 108017. 10.1016/j.jdiacomp.2021.108017 34389235

[B13] Baptista F. I. Pinheiro H. Gomes C. A. Ambrósio A. F. (2019). Impairment of axonal transport in diabetes: focus on the putative mechanisms underlying peripheral and central neuropathies. Mol. Neurobiol. 56 (3), 2202–2210. 10.1007/s12035-018-1227-1 30003516

[B14] Bartáková A. Nováková M. (2021). Secondary metabolites of plants as modulators of endothelium functions. Int. J. Mol. Sci. 22 (5), 2533. 10.3390/ijms22052533 33802468 PMC7959468

[B15] Beeve A. T. Brazill J. M. Scheller E. L. (2019). Peripheral neuropathy as a component of skeletal disease in diabetes. Curr. Osteoporos. Rep. 17 (5), 256–269. 10.1007/s11914-019-00528-8 31392667 PMC6817763

[B16] Berge O. G. (2011). Predictive validity of behavioural animal models for chronic pain. Br. J. Pharmacol. 164 (4), 1195–1206. 10.1111/j.1476-5381.2011.01300.x 21371010 PMC3229757

[B17] Blesneac I. Themistocleous A. C. Fratter C. Conrad L. J. Ramirez J. D. Cox J. J. (2018). Rare NaV1.7 variants associated with painful diabetic peripheral neuropathy. Pain 159 (3), 469–480. 10.1097/j.pain.0000000000001116 29176367 PMC5828379

[B18] Bromberg T. Gasquet N. C. Ricker C. N. Wu C. (2024). Healthcare costs and medical utilization patterns associated with painful and severe painful diabetic peripheral neuropathy. Endocrine 86 (3), 1014–1024. 10.1007/s12020-024-03954-6 39001927 PMC11554691

[B19] Bus S. A. Yang Q. X. Wang J. H. Smith M. B. Wunderlich R. Cavanagh P. R. (2002). Intrinsic muscle atrophy and toe deformity in the diabetic neuropathic foot: a magnetic resonance imaging study. Diabetes Care 25 (8), 1444–1450. 10.2337/diacare.25.8.1444 12145248

[B20] Bus S. A. Maas M. de Lange A. Michels R. P. Levi M. (2005). Elevated plantar pressures in neuropathic diabetic patients with claw/hammer toe deformity. J. Biomech. 38 (9), 1918–1925. 10.1016/j.jbiomech.2004.07.034 16023481

[B21] Cakir L. (2025). Polypharmacy in type 2 diabetes mellitus and related conditions: a double-edged sword: a narrative review. Adv. Clin. Pharmacol. Ther. 2 (2), 39–46. 10.63623/sr9fb890

[B22] Calcutt N. A. (2020). Diabetic neuropathy and neuropathic pain: a (con)fusion of pathogenic mechanisms? Pain 161 (Suppl. 1), S65–s86. 10.1097/j.pain.0000000000001922 32999525 PMC7521457

[B23] Candelario-Jalil E. Taheri S. Yang Y. Sood R. Grossetete M. Estrada E. Y. (2007). Cyclooxygenase inhibition limits blood-brain barrier disruption following intracerebral injection of tumor necrosis factor-alpha in the rat. J. Pharmacol. Exp. Ther. 323 (2), 488–498. 10.1124/jpet.107.127035 17704356

[B24] Catrina S. B. Zheng X. (2021). Hypoxia and hypoxia-inducible factors in diabetes and its complications. Diabetologia 64 (4), 709–716. 10.1007/s00125-021-05380-z 33496820 PMC7940280

[B25] Chandra P. Sachan N. Saraswat N. Vyawahare N. (2024). A detailed review of molecular pathways and mechanisms responsible for the development and aggravation of neuropathy and nephropathy in diabetes. Curr. Mol. Pharmacol. 17 (1), e280323215026. 10.2174/1874467217666230328084215 37018534

[B26] Chen L. Veves A. (2023). Micro- and macrovascular disease in diabetic neuropathy, in Diabetic neuropathy: advances in pathophysiology and clinical management *,* eds. Tesfaye S. Gibbons C. H. Malik R. A. Veves A. (Cham: Springer International Publishing), 351–361.

[B27] Chen T. Li H. Yin Y. Zhang Y. Liu Z. Liu H. (2017). Interactions of Notch1 and TLR4 signaling pathways in DRG neurons of *in vivo* and *in vitro* models of diabetic neuropathy. Sci. Rep. 7 (1), 14923. 10.1038/s41598-017-15053-w 29097792 PMC5668305

[B28] Chen X. Duan Y. Riley A. M. Welch M. A. White F. A. Grant M. B. (2019). Long-term diabetic microenvironment augments the decay rate of capsaicin-induced currents in mouse dorsal root ganglion neurons. Molecules 24 (4), 775. 10.3390/molecules24040775 30795543 PMC6412516

[B29] Chen Q. Yin C. Li Y. Yang Z. Tian Z. (2021). Pharmacokinetic interaction between peimine and paeoniflorin in rats and its potential mechanism. Pharm. Biol. 59 (1), 129–133. 10.1080/13880209.2021.1875013 33721550 PMC7971317

[B30] Chen Y. Lu R. Wang Y. Gan P. (2022). Shaoyao Gancao decoction ameliorates paclitaxel-induced peripheral neuropathy *via* suppressing TRPV1 and TLR4 signaling expression in rats. Drug Des. Devel Ther. 16, 2067–2081. 10.2147/dddt.S357638 35795847 PMC9252300

[B31] Cheng C. Lin J.-z. Li L. Yang J.-l. Jia W.-w. Huang Y.-h. (2016). Pharmacokinetics and disposition of monoterpene glycosides derived from Paeonia lactiflora roots (Chishao) after intravenous dosing of antiseptic XueBiJing injection in human subjects and rats. Acta Pharmacol. Sin. 37 (4), 530–544. 10.1038/aps.2015.103 26838074 PMC4820793

[B32] Cheng Y. C. Chu L. W. Chen J. Y. Hsieh S. L. Chang Y. C. Dai Z. K. (2020). Loganin attenuates high glucose-induced schwann cells pyroptosis by inhibiting ROS generation and NLRP3 inflammasome activation. Cells 9 (9), 1948. 10.3390/cells9091948 32842536 PMC7564733

[B33] Chiang J. C. B. Arnold R. Dhanapalaratnam R. Markoulli M. Krishnan A. V. (2022). Current and emerging pharmacotherapeutic interventions for the treatment of peripheral nerve disorders. Pharm. (Basel) 15 (5), 607. 10.3390/ph15050607 35631433 PMC9144529

[B34] Choi J. W. Im J. H. Balakrishnan R. (2025). Paeoniflorin exercise-mimetic potential regulates the Nrf2/HO-1/BDNF/CREB and APP/BACE-1/NF-κB/MAPK signaling pathways to reduce cognitive impairments and neuroinflammation in amnesic mouse model. Biomed. Pharmacother. 189, 118299. 10.1016/j.biopha.2025.118299 40592207

[B35] Chong Z. Z. Souayah N. (2025). Crumbling pathogenesis and biomarkers for diabetic peripheral neuropathy. Biomedicines 13 (2), 413. 10.3390/biomedicines13020413 40002826 PMC11853266

[B36] Chowdhury S. K. R. Dobrowsky R. T. Fernyhough P. (2011). Nutrient excess and altered mitochondrial proteome and function contribute to neurodegeneration in diabetes. Mitochondrion 11 (6), 845–854. 10.1016/j.mito.2011.06.007 21742060 PMC3375692

[B37] Chu V. Einolf H. J. Evers R. Kumar G. Moore D. Ripp S. (2009). *In vitro* and *in vivo* induction of cytochrome p450: a survey of the current practices and recommendations: a pharmaceutical research and manufacturers of America perspective. Drug Metab. Dispos. 37 (7), 1339–1354. 10.1124/dmd.109.027029 19389860

[B38] Crasto W. Altaf Q. A. Selvaraj D. R. Jack B. Patel V. Nawaz S. (2022). Frequency Rhythmic Electrical Modulation System (FREMS) to alleviate painful diabetic peripheral neuropathy: a pilot, randomised controlled trial (the FREMSTOP study). Diabet. Med. 39 (3), e14710. 10.1111/dme.14710 34605077

[B39] Derouiche I. (2022). How does neurofilament phosphorylation regulate axonal outgrowth and stabilization? Biophysical J. 121 (3), 268a–269a. 10.1016/j.bpj.2021.11.1405

[B40] Di Marco G. S. Reuter S. Hillebrand U. Amler S. König M. Larger E. (2009). The soluble VEGF receptor sFlt1 contributes to endothelial dysfunction in CKD. J. Am. Soc. Nephrol. 20 (10), 2235–2245. 10.1681/asn.2009010061 19608702 PMC2754110

[B41] Ding R. Zhu S. Zhao X. Yue R. (2023). Vascular endothelial growth factor levels in diabetic peripheral neuropathy: a systematic review and meta-analysis. Front. Endocrinol. (Lausanne) 14, 1169405. 10.3389/fendo.2023.1169405 37251664 PMC10213658

[B42] Dou X.-Y. An M. (2025). Advances in the application of platelet-rich plasma in peripheral nerve injuries. Anesthesiol. Perioper. Sci. 3 (2), 19. 10.1007/s44254-025-00100-x

[B44] Du W. Wang N. Li F. Jia K. An J. Liu Y. (2019). STAT3 phosphorylation mediates high glucose-impaired cell autophagy in an HDAC1-dependent and -independent manner in schwann cells of diabetic peripheral neuropathy. Faseb J. 33 (7), 8008–8021. 10.1096/fj.201900127R 30913399

[B45] Eckersley L. (2002). Role of the schwann cell in diabetic neuropathy. Int. Rev. Neurobiol. 50, 293–321. 10.1016/s0074-7742(02)50081-7 12198814

[B46] Ermis N. Gullu H. Caliskan M. Unsal A. Kulaksizoglu M. Muderrisoglu H. (2010). Gabapentin therapy improves heart rate variability in diabetic patients with peripheral neuropathy. J. Diabetes Complicat. 24 (4), 229–233. 10.1016/j.jdiacomp.2008.12.001 19195912

[B47] Estacion M. Harty T. P. Choi J. S. Tyrrell L. Dib-Hajj S. D. Waxman S. G. (2009). A sodium channel gene SCN9A polymorphism that increases nociceptor excitability. Ann. Neurol. 66 (6), 862–866. 10.1002/ana.21895 20033988

[B48] Fan Y. F. Xie Y. Liu L. Ho H. M. Wong Y. F. Liu Z. Q. (2012). Paeoniflorin reduced acute toxicity of Aconitine in rats is associated with the pharmacokinetic alteration of Aconitine. J. Ethnopharmacol. 141 (2), 701–708. 10.1016/j.jep.2011.09.005 21930193

[B49] Fan B. Chopp M. Zhang Z. G. Liu X. S. (2020). Emerging roles of microRNAs as biomarkers and therapeutic targets for diabetic neuropathy. Front. Neurology 11, 558758. 10.3389/fneur.2020.558758 33192992 PMC7642849

[B50] Fan J. Jeffery M. M. Hooten W. M. Shah N. D. McCoy R. G. (2021). Trends in pain medication initiation among patients with newly diagnosed diabetic peripheral neuropathy, 2014-2018. JAMA Netw. Open 4 (1), e2035632. 10.1001/jamanetworkopen.2020.35632 33507254 PMC7844595

[B51] Fang F. Wang J. Wang Y. F. Peng Y. D. (2018). Microangiopathy in diabetic polyneuropathy revisited. Eur. Rev. Med. Pharmacol. Sci. 22 (19), 6456–6462. 10.26355/eurrev_201810_16058 30338814

[B52] Farhad K. (2019). Current diagnosis and treatment of painful small fiber neuropathy. Curr. Neurology Neurosci. Rep. 19 (12), 103. 10.1007/s11910-019-1020-1 31773305

[B53] Fei F. Yang H. Peng Y. Wang P. Wang S. Zhao Y. (2016). Sensitive analysis and pharmacokinetic study of the isomers paeoniflorin and albiflorin after oral administration of total glucosides of white paeony capsule in rats. J. Chromatogr. B Anal. Technol. Biomed. Life Sci. 1022, 30–37. 10.1016/j.jchromb.2016.04.005 27070118

[B54] Feldman E. L. Callaghan B. C. Pop-Busui R. Zochodne D. W. Wright D. E. Bennett D. L. (2019). Diabetic neuropathy. Nat. Rev. Dis. Prim. 5 (1), 41. 10.1038/s41572-019-0092-1 31197153

[B55] Feng Y. Chen L. Luo Q. Wu M. Chen Y. Shi X. (2018). Involvement of microRNA-146a in diabetic peripheral neuropathy through the regulation of inflammation. Drug Des. Devel Ther. 12, 171–177. 10.2147/dddt.S157109 29398906 PMC5775734

[B56] Freeman R. Durso-DeCruz E. Emir B. (2008). Efficacy, safety, and tolerability of pregabalin treatment for painful diabetic peripheral neuropathy: findings from seven randomized, controlled trials across a range of doses. Diabetes Care 31 (7), 1448–1454. 10.2337/dc07-2105 18356405 PMC2453685

[B57] Galiero R. Caturano A. Vetrano E. Beccia D. Brin C. Alfano M. (2023). Peripheral neuropathy in diabetes mellitus: pathogenetic mechanisms and diagnostic options. Int. J. Mol. Sci. 24 (4), 3554. 10.3390/ijms24043554 36834971 PMC9967934

[B58] Gao L.-N. Zhang Y. Cui Y.-L. Akinyi O. M. (2015). Comparison of paeoniflorin and albiflorin on human CYP3A4 and CYP2D6. Evidence-Based Complementary Altern. Med. 2015 (1), 470219. 10.1155/2015/470219 26089940 PMC4452296

[B59] Gao N. Li M. Wang W. Liu Z. Guo Y. (2024). The dual role of TRPV1 in peripheral neuropathic pain: pain switches caused by its sensitization or desensitization. Front. Mol. Neurosci. 17, 1400118. 10.3389/fnmol.2024.1400118 39315294 PMC11417043

[B60] Gogan A. Potre O. Avram V. F. Andor M. Caruntu F. Timar B. (2025). Cardiac autonomic neuropathy in diabetes mellitus: pathogenesis, epidemiology, diagnosis and clinical implications: a narrative review. J. Clin. Med. 14 (3), 671. 10.3390/jcm14030671 39941342 PMC11818907

[B61] Goldstein D. J. Lu Y. Detke M. J. Lee T. C. Iyengar S. (2005). Duloxetine vs. placebo in patients with painful diabetic neuropathy. PAIN 116 (1), 109–118. 10.1016/j.pain.2005.03.029 15927394

[B62] Gorczyca-Siudak D. Dziemidok P. (2022). The 8-Week efficacy of Frequency Rhythmic Electrical Modulated System (FREMS) as an Add-on therapy in the treatment of symptomatic diabetic peripheral polyneuropathy. Int. J. Environ. Res. Public Health 20 (1), 111. 10.3390/ijerph20010111 36612433 PMC9819549

[B63] Gordois A. Scuffham P. Shearer A. Oglesby A. Tobian J. A. (2003). The health care costs of diabetic peripheral neuropathy in the U.S. Diabetes Care 26 (6), 1790–1795. 10.2337/diacare.26.6.1790 12766111

[B64] Gordon S. Uazman A. Dinesh S. Solomon T. (2022). The treatment of painful diabetic neuropathy. Curr. Diabetes Rev. 18 (5), 42–96. 10.2174/1573399817666210707112413 34238163

[B65] Griffin J. M. Fackelmeier B. Clemett C. A. Fong D. M. Mouravlev A. Young D. (2020). Astrocyte-selective AAV-ADAMTS4 gene therapy combined with hindlimb rehabilitation promotes functional recovery after spinal cord injury. Exp. Neurol. 327, 113232. 10.1016/j.expneurol.2020.113232 32044329

[B66] Guo K. Eid S. A. Elzinga S. E. Pacut C. Feldman E. L. Hur J. (2020). Genome-wide profiling of DNA methylation and gene expression identifies candidate genes for human diabetic neuropathy. Clin. Epigenetics 12 (1), 123. 10.1186/s13148-020-00913-6 32787975 PMC7425575

[B67] Ha H. Lee J. K. Lee H. Y. Koh W. S. Seo C. S. Lee M. Y. (2011). Safety evaluation of Yukmijihwang-Tang: assessment of acute and subchronic toxicity in rats. Evid. Based Complement. Altern. Med. 2011, 672136. 10.1155/2011/672136 21234385 PMC3017901

[B68] Hanani M. Spray D. C. (2020). Emerging importance of satellite glia in nervous system function and dysfunction. Nat. Rev. Neurosci. 21 (9), 485–498. 10.1038/s41583-020-0333-z 32699292 PMC7374656

[B69] Hao W. Tashiro S. Hasegawa T. Sato Y. Kobayashi T. Tando T. (2015). Hyperglycemia promotes schwann cell De-differentiation and De-myelination *via* sorbitol accumulation and Igf1 protein down-regulation. J. Biol. Chem. 290 (28), 17106–17115. 10.1074/jbc.M114.631291 25998127 PMC4498049

[B70] Hazarika S. Dokun A. O. Li Y. Popel A. S. Kontos C. D. Annex B. H. (2007). Impaired angiogenesis after Hindlimb ischemia in type 2 diabetes mellitus. Circulation Res. 101 (9), 948–956. 10.1161/CIRCRESAHA.107.160630 17823371

[B71] Hedayat A. Chandrasekaran K. Zilliox L. A. Russell J. W. (2023). Targeting the mitochondrion in diabetic neuropathy, in Diabetic neuropathy: advances in pathophysiology and clinical management *,* eds. Tesfaye S. Gibbons C. H. Malik R. A. Veves A. (Cham: Springer International Publishing), 307–326.

[B72] Heinrich M. Jalil B. Abdel-Tawab M. Echeverria J. Kulić Ž. McGaw L. J. (2022). Best practice in the chemical characterisation of extracts used in pharmacological and toxicological research-The ConPhyMP-Guidelines. Front. Pharmacol. 13, 953205. 10.3389/fphar.2022.953205 36176427 PMC9514875

[B73] Hernandez-Reyes M. Oo T. T. (2025). From receptor to response: dissecting the TLR4 pathway in diabetic neuropathy. Inflammopharmacology 33 (5), 2523–2535. 10.1007/s10787-025-01774-2 40347407

[B74] Herron C. Hastings C. L. Herron-Rice C. Kelly H. M. O'Dwyer J. Duffy G. P. (2021). A thermoresponsive Chitosan/β-Glycerophosphate hydrogel for minimally invasive treatment of critical limb ischaemia. Polym. (Basel) 13 (20), 3568. 10.3390/polym13203568 34685327 PMC8539345

[B75] Hersini K. J. Melgaard L. Gazerani P. Petersen L. J. (2014). Microdialysis of inflammatory mediators in the skin: a review. Acta Derm. Venereol. 94 (5), 501–511. 10.2340/00015555-1878 24890140

[B76] Hoeijmakers J. G. Faber C. G. Lauria G. Merkies I. S. Waxman S. G. (2012). Small-fibre neuropathies—advances in diagnosis, pathophysiology and management. Nat. Rev. Neurol. 8 (7), 369–379. 10.1038/nrneurol.2012.97 22641108

[B77] Holtkamp M. Meierkord H. (2007). Anticonvulsant, antiepileptogenic, and antiictogenic pharmacostrategies. Cell Mol. Life Sci. 64 (15), 2023–2041. 10.1007/s00018-007-7021-2 17514360 PMC11136249

[B78] Hong H. Lu X. Wu C. Chen J. Chen C. Zhang J. (2022). A review for the pharmacological effects of paeoniflorin in the nervous system. Front. Pharmacol. 13, 898955. 10.3389/fphar.2022.898955 36046834 PMC9420976

[B79] Hooijmans C. R. Rovers M. M. de Vries R. B. Leenaars M. Ritskes-Hoitinga M. Langendam M. W. (2014). SYRCLE's risk of bias tool for animal studies. BMC Med. Res. Methodol. 14, 43. 10.1186/1471-2288-14-43 24667063 PMC4230647

[B80] Hu F. Lin J. Xiong L. Li Z. Liu W. K. Zheng Y. J. (2024). Exploring the molecular mechanism of Xuebifang in the treatment of diabetic peripheral neuropathy based on bioinformatics and network pharmacology. Front. Endocrinol. (Lausanne) 15, 1275816. 10.3389/fendo.2024.1275816 38390212 PMC10881818

[B81] Huang S. M. Zhang L. Giacomini K. M. (2010). The international transporter consortium: a collaborative group of scientists from academia, industry, and the FDA. Clin. Pharmacol. Ther. 87 (1), 32–36. 10.1038/clpt.2009.236 20019700

[B82] Huang H. Li Y. Huang Q. Lei R. Zou W. Zheng Y. (2021). Efficacy of compound Danshen dripping pills combined with western medicine in the treatment of diabetic retinopathy: a systematic review and meta-analysis of randomized controlled trials. Ann. Palliat. Med. 10 (10), 10954–10962. 10.21037/apm-21-2563 34763458

[B83] Hur J. Sullivan K. A. Callaghan B. C. Pop-Busui R. Feldman E. L. (2013). Identification of factors associated with sural nerve regeneration and degeneration in diabetic neuropathy. Diabetes Care 36 (12), 4043–4049. 10.2337/dc12-2530 24101696 PMC3836098

[B84] Ineichen B. V. Furrer E. Grüninger S. L. Zürrer W. E. Macleod M. R. (2024). Analysis of animal-to-human translation shows that only 5% of animal-tested therapeutic interventions obtain regulatory approval for human applications. PLoS Biol. 22 (6), e3002667. 10.1371/journal.pbio.3002667 38870090 PMC11175415

[B85] Iqbal Z. Azmi S. Yadav R. Ferdousi M. Kumar M. Cuthbertson D. J. (2018). Diabetic peripheral neuropathy: epidemiology, diagnosis, and pharmacotherapy. Clin. Ther. 40 (6), 828–849. 10.1016/j.clinthera.2018.04.001 29709457

[B86] Jang H. N. Oh T. J. (2023). Pharmacological and nonpharmacological treatments for painful diabetic peripheral neuropathy. Diabetes Metab. J. 47 (6), 743–756. 10.4093/dmj.2023.0018 37670573 PMC10695723

[B87] Javed S. Alam U. Malik R. A. (2018). Mirogabalin and emerging therapies for diabetic neuropathy. J. Pain Res. 11, 1559–1566. 10.2147/jpr.S145999 30174455 PMC6110292

[B88] Jeong S. J. Seo C. S. Huh J. I. Shin H. K. (2014). Subacute oral toxicity of Yukmijiwhang-Tang in Crl:CD sprague-dawley rats and its cytotoxicity. Evid. Based Complement. Altern. Med. 2014, 362573. 10.1155/2014/362573 25431608 PMC4238173

[B89] Jiao F. Varghese K. Wang S. Liu Y. Yu H. Booz G. W. (2021). Recent insights into the protective mechanisms of paeoniflorin in neurological, cardiovascular, and renal diseases. J. Cardiovasc Pharmacol. 77 (6), 728–734. 10.1097/fjc.0000000000001021 34001724 PMC8169546

[B90] Jourova L. Anzenbacher P. Anzenbacherova E. (2016). Human gut microbiota plays a role in the metabolism of drugs. Biomed. Pap. Med. Fac. Univ. Palacky. Olomouc Czech Repub. 160 (3), 317–326. 10.5507/bp.2016.039 27485182

[B91] Kajiwara N. Sasaki T. Bradding P. Cruse G. Sagara H. Ohmori K. (2010). Activation of human mast cells through the platelet-activating factor receptor. J. Allergy Clin. Immunol. 125 (5), 1137–1145.e1136. 10.1016/j.jaci.2010.01.056 20392487

[B92] Kang X. F. Lu X. L. Bi C. F. Hu X. D. Li Y. Li J. K. (2023). Xuebijing injection protects sepsis induced myocardial injury by mediating TLR4/NF-κB/IKKα and JAK2/STAT3 signaling pathways. Aging (Albany NY) 15 (16), 8501–8517. 10.18632/aging.204990 37650558 PMC10496990

[B93] Kato H. Miyazaki M. Takeuchi M. Tsukuura H. Sugishita M. Noda Y. (2015). A retrospective study to identify risk factors for somnolence and dizziness in patients treated with pregabalin. J. Pharm. Health Care Sci. 1 (1), 22. 10.1186/s40780-015-0022-7 26819733 PMC4728751

[B94] Kelley M. A. Hackshaw K. V. (2021). Intraepidermal nerve fiber density as measured by skin punch biopsy as a marker for small fiber neuropathy: application in patients with fibromyalgia. Diagn. (Basel) 11 (3), 536. 10.3390/diagnostics11030536 33802768 PMC8002511

[B95] Kempler P. Várkonyi T. Körei A. E. Horváth V. J. (2016). Gastrointestinal autonomic neuropathy in diabetes: the unattended borderline between diabetology and gastroenterology. Diabetologia 59 (3), 401–403. 10.1007/s00125-015-3826-y 26638001

[B96] Kong L. Li J. Yang Y. Tang H. Zou H. (2022). Paeoniflorin alleviates the progression of retinal vein occlusion *via* inhibiting hypoxia inducible factor-1α/vascular endothelial growth factor/STAT3 pathway. Bioengineered 13 (5), 13622–13631. 10.1080/21655979.2022.2081755 35653799 PMC9275925

[B97] Krabbe K. S. Nielsen A. R. Krogh-Madsen R. Plomgaard P. Rasmussen P. Erikstrup C. (2007). Brain-derived neurotrophic factor (BDNF) and type 2 diabetes. Diabetologia 50 (2), 431–438. 10.1007/s00125-006-0537-4 17151862

[B98] Kwiatkowska K. M. Garagnani P. Bonafé M. Bacalini M. G. Sala C. Castellani G. (2025). High-resolution whole-genome DNA methylation revealed unique signatures of painful diabetic neuropathy. Diabetes 74 (4), 640–650. 10.2337/db24-0930 39774670 PMC11926268

[B99] Lam D. Momeni Z. Theaker M. Jagadeeshan S. Yamamoto Y. Ianowski J. P. (2018). RAGE-dependent potentiation of TRPV1 currents in sensory neurons exposed to high glucose. PLOS ONE 13 (2), e0193312. 10.1371/journal.pone.0193312 29474476 PMC5825096

[B100] Langley B. Gensert J. M. Beal M. F. Ratan R. R. (2005). Remodeling chromatin and stress resistance in the central nervous system: histone deacetylase inhibitors as novel and broadly effective neuroprotective agents. Curr. Drug Targets CNS Neurol. Disord. 4 (1), 41–50. 10.2174/1568007053005091 15723612

[B101] Li Q. S. Cheng P. Favis R. Wickenden A. Romano G. Wang H. (2015). SCN9A variants may be implicated in neuropathic pain associated with diabetic peripheral neuropathy and pain severity. Clin. J. Pain 31 (11), 976–982. 10.1097/ajp.0000000000000205 25585270 PMC4894774

[B102] Li L. Yu T. Yu L. Li H. Liu Y. Wang D. (2016). Exogenous brain-derived neurotrophic factor relieves pain symptoms of diabetic rats by reducing excitability of dorsal root ganglion neurons. Int. J. Neurosci. 126 (8), 749–758. 10.3109/00207454.2015.1057725 26441011

[B103] Li H. Jiao Y. Xie M. (2017). Paeoniflorin ameliorates atherosclerosis by suppressing TLR4-Mediated NF-κB activation. Inflammation 40 (6), 2042–2051. 10.1007/s10753-017-0644-z 28791506

[B104] Li B. Yang Z. B. Lei S. S. Su J. Jin Z. W. Chen S. H. (2018). Combined antihypertensive effect of paeoniflorin enriched extract and metoprolol in spontaneously hypertensive rats. Pharmacogn. Mag. 14 (53), 44–52. 10.4103/pm.pm_483_16 29576700 PMC5858241

[B105] Li Y. S. Kawasaki Y. Watanabe S. Ootsuyama Y. Kasai H. Kawai K. (2021). Diurnal and day-to-day variation of urinary oxidative stress marker 8-hydroxy-2'-deoxyguanosine. J. Clin. Biochem. Nutr. 68 (1), 18–22. 10.3164/jcbn.19-105 33536708 PMC7844656

[B106] Li J. Yu M. Fu S. Liu D. Tan Y. (2022). Role of selective histone deacetylase 6 inhibitor ACY-1215 in cancer and other human diseases. Front. Pharmacol. 13. 907981, 10.3389/fphar.2022.907981 35652048 PMC9149003

[B107] Liu H. Sahi J. (2025). Peripheral nerve barrier and its implications for drug delivery. Drug Discov. Today 30 (9), 104435. 10.1016/j.drudis.2025.104435 40712925

[B108] Liu Z. Q. Zhou H. Liu L. Jiang Z. H. Wong Y. F. Xie Y. (2005). Influence of co-administrated sinomenine on pharmacokinetic fate of paeoniflorin in unrestrained conscious rats. J. Ethnopharmacol. 99 (1), 61–67. 10.1016/j.jep.2005.01.052 15848021

[B109] Liu Z. Q. Jiang Z. H. Liu L. Hu M. (2006). Mechanisms responsible for poor oral bioavailability of paeoniflorin: role of intestinal disposition and interactions with sinomenine. Pharm. Res. 23 (12), 2768–2780. 10.1007/s11095-006-9100-8 17063398

[B110] Liu X. S. Fan B. Szalad A. Jia L. Wang L. Wang X. (2017). MicroRNA-146a mimics reduce the peripheral neuropathy in type 2 diabetic mice. Diabetes 66 (12), 3111–3121. 10.2337/db16-1182 28899883 PMC5697943

[B111] Liu H. Chen Y. Huang L. Sun X. Fu T. Wu S. (2018). Drug distribution into peripheral nerve. J. Pharmacol. Exp. Ther. 365 (2), 336–345. 10.1124/jpet.117.245613 29511033

[B112] Liu S. C. Hu W. Y. Zhang W. Y. Yang L. Li Y. Xiao Z. C. (2019). Paeoniflorin attenuates impairment of spatial learning and hippocampal long-term potentiation in mice subjected to chronic unpredictable mild stress. Psychopharmacol. Berl. 236 (9), 2823–2834. 10.1007/s00213-019-05257-5 31115613

[B113] Liu Y. Ming Z. Jia S. Su Y. Wu K. Yan W. (2025). Multi-omics insights into the gut-spinal cord axis: paeoniflorin mitigates diabetic neuropathic pain in rats through microbial-metabolic crosstalk. Food Sci. Hum. Wellness. 10.26599/FSHW.2025.9250837

[B114] Liu Z. Lu J. Sha W. Lei T. (2025). Comprehensive treatment of diabetic endothelial dysfunction based on pathophysiological mechanism. Front. Med. 12 1509884, 10.3389/fmed.2025.1509884 40093018 PMC11906411

[B115] Llorián-Salvador M. Cabeza-Fernández S. Gomez-Sanchez J. A. de la Fuente A. G. (2024). Glial cell alterations in diabetes-induced neurodegeneration. Cell Mol. Life Sci. 81 (1), 47. 10.1007/s00018-023-05024-y 38236305 PMC10796438

[B116] Long M. Hu Z. Long F. Chen Y. Liu L. Wang M. (2025). The capsaicin receptor TRPV1 reduces sepsis-associated brain injury in mice by inhibiting pyroptosis. Anesthesiol. Perioper. Sci. 3 (3), 33. 10.1007/s44254-025-00115-4

[B117] Low P. A. Lagerlund T. D. McManis P. G. (1989). Nerve blood flow and oxygen delivery in normal, diabetic, and ischemic neuropathy, in International review of neurobiology *,* eds. Smythies J.R. Bradley R.J. Academic Press), 355–438. 10.1016/s0074-7742(08)60283-4 2557297

[B118] Lux T. J. Hu X. Ben-Kraiem A. Blum R. Chen J. T. Rittner H. L. (2019). Regional differences in tight junction protein expression in the Blood-DRG barrier and their alterations after nerve traumatic injury in rats. Int. J. Mol. Sci. 21 (1), 270. 10.3390/ijms21010270 31906086 PMC6981987

[B119] Maher R. Moreno-Borrallo A. Jindal D. Mai B. T. Ruiz-Hernandez E. Harkin A. (2023). Intranasal polymeric and lipid-based nanocarriers for CNS drug delivery. Pharmaceutics 15 (3), 746. 10.3390/pharmaceutics15030746 36986607 PMC10051709

[B120] Malik R. A. Tesfaye S. Thompson S. D. Veves A. Sharma A. K. Boulton A. J. M. (1993). Endoneurial localisation of microvascular damage in human diabetic neuropathy. Diabetologia 36 (5), 454–459. 10.1007/BF00402283 8314451

[B121] Martignoni M. Groothuis G. M. de Kanter R. (2006). Species differences between mouse, rat, dog, monkey and human CYP-mediated drug metabolism, inhibition and induction. Expert Opin. Drug Metab. Toxicol. 2 (6), 875–894. 10.1517/17425255.2.6.875 17125407

[B122] McGuire J. F. Rouen S. Siegfreid E. Wright D. E. Dobrowsky R. T. (2009). Caveolin-1 and altered neuregulin signaling contribute to the pathophysiological progression of diabetic peripheral neuropathy. Diabetes 58 (11), 2677–2686. 10.2337/db09-0594 19675140 PMC2768162

[B123] Meng P. Zhang X. Liu T. T. Liu J. Luo Y. Xie M. X. (2023). A whole transcriptome profiling analysis for antidepressant mechanism of Xiaoyaosan mediated synapse loss *via* BDNF/trkB/PI3K signal axis in CUMS rats. BMC Complement. Med. Ther. 23 (1), 198. 10.1186/s12906-023-04000-0 37322430 PMC10273699

[B124] Middlemas A. Delcroix J. D. Sayers N. M. Tomlinson D. R. Fernyhough P. (2003). Enhanced activation of axonally transported stress-activated protein kinases in peripheral nerve in diabetic neuropathy is prevented by neurotrophin-3. Brain 126 (Pt 7), 1671–1682. 10.1093/brain/awg150 12805110

[B125] Mizisin A. P. (2014). Mechanisms of diabetic neuropathy: Schwann cells. Handb. Clin. Neurol. 126, 401–428. 10.1016/b978-0-444-53480-4.00029-1 25410236

[B126] Morozova N. Avramovič M. Z. Markelj G. Toplak N. Avčin T. (2024). Dynamics of serum levels of TNF-α in a longitudinal follow-up study in 98 patients with juvenile idiopathic arthritis treated with anti-TNF-α biological drugs. Clin. Rheumatol. 43 (7), 2287–2293. 10.1007/s10067-024-07012-4 38775868 PMC11189329

[B127] Nashtahosseini Z. Eslami M. Paraandavaji E. Haraj A. Dowlat B. F. Hosseinzadeh E. (2025). Cytokine signaling in diabetic neuropathy: a key player in peripheral nerve damage. Biomedicines 13 (3), 589. 10.3390/biomedicines13030589 40149566 PMC11940495

[B128] Obata H. (2017). Analgesic mechanisms of antidepressants for neuropathic pain. Int. J. Mol. Sci. 18 (11), 2483. 10.3390/ijms18112483 29160850 PMC5713449

[B129] Ohishi A. Chisaki Y. Hira D. Nagasawa K. Terada T. (2015). Opioid analgesics increase incidence of somnolence and dizziness as adverse effects of pregabalin: a retrospective study. J. Pharm. Health Care Sci. 1 (1), 30. 10.1186/s40780-015-0032-5 26819741 PMC4729150

[B130] Osorio F. G. Soria-Valles C. Santiago-Fernández O. Freije J. M. P. López-Otín C. (2016). Chapter four - nf-κb signaling as a driver of ageing, in International review of cell and molecular biology *,* eds. Jeon K.W. Galluzzi L. Academic Press), 133–174. 10.1016/bs.ircmb.2016.04.003 27572128

[B131] Ou X. Yu Z. Pan C. Zheng X. Li D. Qiao Z. (2025). Paeoniflorin: a review of its pharmacology, pharmacokinetics and toxicity in diabetes. Front. Pharmacol. 16, 1551368. 10.3389/fphar.2025.1551368 40260393 PMC12009869

[B132] Paisey R. B. Darby T. George A. M. Waterson M. Hewson P. Paisey C. F. (2016). Prediction of protective sensory loss, neuropathy and foot ulceration in type 2 diabetes. BMJ Open Diabetes Res. Care 4 (1), e000163. 10.1136/bmjdrc-2015-000163 27239314 PMC4873950

[B133] Pan P. Dobrowsky R. T. (2013). Differential expression of neuregulin-1 isoforms and downregulation of erbin are associated with Erb B2 receptor activation in diabetic peripheral neuropathy. Acta Neuropathol. Commun. 1 (1), 39. 10.1186/2051-5960-1-39 24252174 PMC3893607

[B134] Park K. Kim S. Ko Y.-J. Park B.-J. (2020). Duloxetine and cardiovascular adverse events: a systematic review and meta-analysis. J. Psychiatric Res. 124, 109–114. 10.1016/j.jpsychires.2020.02.022 32135389

[B135] Picci C. Wong V. S. C. Costa C. J. McKinnon M. C. Goldberg D. C. Swift M. (2020). HDAC6 inhibition promotes α-tubulin acetylation and ameliorates CMT2A peripheral neuropathy in mice. Exp. Neurol. 328, 113281. 10.1016/j.expneurol.2020.113281 32147437

[B136] Pop-Busui R. Ang L. Boulton A. J. M. Feldman E. L. Marcus R. L. Mizokami-Stout K. (2022). ADA clinical compendia series, in Diagnosis and treatment of painful diabetic peripheral neuropathy. (Arlington (VA): American Diabetes Association. 35544662

[B137] Preisner F. Behnisch R. Schwehr V. Godel T. Schwarz D. Foesleitner O. (2022). Quantitative MR-Neurography at 3.0T: inter-scanner reproducibility. Front. Neurosci. 16, 16 10.3389/fnins.2022.817316 35250457 PMC8888927

[B138] Prior R. Van Helleputte L. Klingl Y. E. Van Den Bosch L. (2018). HDAC6 as a potential therapeutic target for peripheral nerve disorders. Expert Opin. Ther. Targets 22 (12), 993–1007. 10.1080/14728222.2018.1541235 30360671

[B139] Quilici S. Chancellor J. Löthgren M. Simon D. Said G. Le T. K. (2009). Meta-analysis of duloxetine vs. pregabalin and gabapentin in the treatment of diabetic peripheral neuropathic pain. BMC Neurol. 9 (1), 6. 10.1186/1471-2377-9-6 19208243 PMC2663537

[B140] Rahmanian-Devin P. Baradaran Rahimi V. Askari V. R. (2021). Thermosensitive Chitosan-β-Glycerophosphate hydrogels as targeted drug delivery systems: an overview on preparation and their applications. Adv. Pharmacol. Pharm. Sci. 2021, 6640893. 10.1155/2021/6640893 34036263 PMC8116164

[B141] Raouf Y. S. (2024). Targeting histone deacetylases: emerging applications beyond cancer. Drug Discov. Today 29 (9), 104094. 10.1016/j.drudis.2024.104094 38997001

[B142] Reeves N. D. Orlando G. Brown S. J. (2021). Sensory-motor mechanisms increasing falls risk in diabetic peripheral neuropathy. Med. Kaunas. 57 (5), 457. 10.3390/medicina57050457 34066681 PMC8150714

[B143] Richter R. W. Portenoy R. Sharma U. Lamoreaux L. Bockbrader H. Knapp L. E. (2005). Relief of painful diabetic peripheral neuropathy with pregabalin: a randomized, placebo-controlled trial. J. Pain 6 (4), 253–260. 10.1016/j.jpain.2004.12.007 15820913

[B144] Rudofsky G. Jr. Reismann P. Witte S. Humpert P. M. Isermann B. Chavakis T. (2004). Asp299Gly and Thr399Ile genotypes of the TLR4 gene are associated with a reduced prevalence of diabetic neuropathy in patients with type 2 diabetes. Diabetes Care 27 (1), 179–183. 10.2337/diacare.27.1.179 14693986

[B145] Sadosky A. Schaefer C. Tölle T. Siffert J. Dukes E. (2005). Impact of painful Diabetic Peripheral Neuropathy (DPN) on pain management, employment status, work productivity and health resource utilization: a survey of six European countries. J. Pain 6 (3), S73. 10.1016/j.jpain.2005.01.288

[B146] Sadosky A. Mardekian J. Parsons B. Hopps M. Bienen E. J. Markman J. (2015). Healthcare utilization and costs in diabetes relative to the clinical spectrum of painful diabetic peripheral neuropathy. J. Diabetes Its Complicat. 29 (2), 212–217. 10.1016/j.jdiacomp.2014.10.013 25498300

[B147] Satapathy P. Gaidhane A. M. Vadia N. Menon S. V. Chennakesavulu K. Panigrahi R. (2025). Prevalence of polypharmacy among older adults with diabetes: a systematic review and meta-analysis. Aging Clin. Exp. Res. 37 (1), 335. 10.1007/s40520-025-03240-z 41296133 PMC12657567

[B148] Schmeichel A. M. Schmelzer J. D. Low P. A. (2003). Oxidative injury and apoptosis of dorsal root ganglion neurons in chronic experimental diabetic neuropathy. Diabetes 52 (1), 165–171. 10.2337/diabetes.52.1.165 12502508

[B149] Sen A. Mohanraj P. S. Ranjan A. Rajendran V. ArulVijayaVani S. Balan Y. (2023). Unraveling the role of tumor necrosis factor-alpha in diabetic peripheral neuropathy: a systematic review and meta-analysis. Cureus 15 (12), e49926. 10.7759/cureus.49926 38179375 PMC10764202

[B150] Serhiyenko V. A. Serhiyenko A. A. (2018). Cardiac autonomic neuropathy: risk factors, diagnosis and treatment. World J. Diabetes 9 (1), 1–24. 10.4239/wjd.v9.i1.1 29359025 PMC5763036

[B151] Shao Y.-X. Gong Q. Qi X.-M. Wang K. Wu Y.-g. (2019). Paeoniflorin ameliorates macrophage infiltration and activation by inhibiting the TLR4 signaling pathway in diabetic nephropathy. Front. Pharmacol. 10. 566. 10.3389/fphar.2019.00566 31191309 PMC6540689

[B152] Sima A. A. F. Zhang W. (2014). Chapter 28 - mechanisms of diabetic neuropathy: axon dysfunction, in Handbook of clinical neurology *,* eds. Zochodne D. W. Malik R. A. Elsevier), 429–442. 10.1016/B978-0-444-53480-4.00031-X 25410237

[B153] Singh K. Singh V. K. Agrawal N. K. Gupta S. K. Singh K. (2013). Association of Toll-like receptor 4 polymorphisms with diabetic foot ulcers and application of artificial neural network in DFU risk assessment in type 2 diabetes patients. Biomed. Res. Int. 2013, 318686. 10.1155/2013/318686 23936790 PMC3725976

[B154] Sollmann N. Weidlich D. Klupp E. Cervantes B. Ganter C. Zimmer C. (2020). T2 mapping of the distal sciatic nerve in healthy subjects and patients suffering from lumbar disc herniation with nerve compression. Magnetic Reson. Mater. Phys. Biol. Med. 33 (5), 713–724. 10.1007/s10334-020-00832-w 32048099 PMC7502059

[B155] Song S. Xiao X. Guo D. Mo L. Bu C. Ye W. (2017). Protective effects of Paeoniflorin against AOPP-induced oxidative injury in HUVECs by blocking the ROS-HIF-1α/VEGF pathway. Phytomedicine 34, 115–126. 10.1016/j.phymed.2017.08.010 28899493

[B156] Stirban A. Gawlowski T. Roden M. (2014). Vascular effects of advanced glycation endproducts: clinical effects and molecular mechanisms. Mol. Metab. 3 (2), 94–108. 10.1016/j.molmet.2013.11.006 24634815 PMC3953708

[B157] Štuhec M. (2013). Duloxetine-induced life-threatening long QT syndrome. Wien. Klinische Wochenschr. 125 (5), 165–166. 10.1007/s00508-013-0330-6 23440523

[B158] Sullivan K. A. Hayes J. M. Wiggin T. D. Backus C. Su Oh S. Lentz S. I. (2007). Mouse models of diabetic neuropathy. Neurobiol. Dis. 28 (3), 276–285. 10.1016/j.nbd.2007.07.022 17804249 PMC3730836

[B159] Sun H. Saeedi P. Karuranga S. Pinkepank M. Ogurtsova K. Duncan B. B. (2022). IDF diabetes atlas: global, regional and country-level diabetes prevalence estimates for 2021 and projections for 2045. Diabetes Res. Clin. Pract. 183, 109119. 10.1016/j.diabres.2021.109119 34879977 PMC11057359

[B160] Taiana M. M. Lombardi R. Porretta-Serapiglia C. Ciusani E. Oggioni N. Sassone J. (2014). Neutralization of schwann cell-secreted VEGF is protective to *in vitro* and *in vivo* experimental diabetic neuropathy. PLoS One 9 (9), e108403. 10.1371/journal.pone.0108403 25268360 PMC4182455

[B161] Tajabadi Z. Dadkhah P. A. Gholami Chahkand M. S. Esmaeilpour Moallem F. Karimi M. A. Amini-Salehi E. (2025). Exploring the role of exosomes in diabetic neuropathy: from molecular mechanisms to therapeutic potential. Biomed. Pharmacother. 185, 117959. 10.1016/j.biopha.2025.117959 40056828

[B162] Tavakkoli A. Iranshahi M. Hasheminezhad S. H. Hayes A. W. Karimi G. (2019). The neuroprotective activities of natural products through the Nrf2 upregulation. Phytother. Res. 33 (9), 2256–2273. 10.1002/ptr.6427 31322315

[B163] Terkelsen A. J. Karlsson P. Lauria G. Freeman R. Finnerup N. B. Jensen T. S. (2017). The diagnostic challenge of small fibre neuropathy: clinical presentations, evaluations, and causes. Lancet Neurology 16 (11), 934–944. 10.1016/S1474-4422(17)30329-0 29029847

[B164] Tesfaye S. Kempler P. (2023). Conventional management and current guidelines for painful diabetic neuropathy. Diabetes Res. Clin. Pract. 206 (Suppl. 1), 110765. 10.1016/j.diabres.2023.110765 38245323

[B165] Tesfaye S. Sloan G. Petrie J. White D. Bradburn M. Julious S. (2022). Comparison of amitriptyline supplemented with pregabalin, pregabalin supplemented with amitriptyline, and duloxetine supplemented with pregabalin for the treatment of diabetic peripheral neuropathic pain (OPTION-DM): a multicentre, double-blind, randomised crossover trial. Lancet 400 (10353), 680–690. 10.1016/S0140-6736(22)01472-6 36007534 PMC9418415

[B166] Thacker M. A. Clark A. K. Bishop T. Grist J. Yip P. K. Moon L. D. F. (2009). CCL2 is a key mediator of microglia activation in neuropathic pain states. Eur. J. Pain 13 (3), 263–272. 10.1016/j.ejpain.2008.04.017 18554968

[B167] Thakur V. Gonzalez M. A. Parada M. Martinez R. D. Chattopadhyay M. (2024). Role of histone deacetylase inhibitor in diabetic painful neuropathy. Mol. Neurobiol. 61 (4), 2283–2296. 10.1007/s12035-023-03701-4 37875708

[B168] Tian M. M. Li Y. X. Liu S. Zhu C. H. Lan X. B. Du J. (2021). Glycosides for peripheral neuropathic pain: a potential medicinal components. Molecules 27 (1), 255. 10.3390/molecules27010255 35011486 PMC8746348

[B169] Tramullas M. Francés R. de la Fuente R. Velategui S. Carcelén M. García R. (2018). MicroRNA-30c-5p modulates neuropathic pain in rodents. Sci. Transl. Med. 10 (453), eaao6299. 10.1126/scitranslmed.aao6299 30089634

[B170] Tyagi S. Higerd-Rusli G. P. Ghovanloo M. R. Dib-Hajj F. Zhao P. Liu S. (2024). Compartment-specific regulation of Na(V)1.7 in sensory neurons after acute exposure to TNF-α. Cell Rep. 43 (2), 113685. 10.1016/j.celrep.2024.113685 38261513 PMC10947185

[B171] Vinik A. I. Maser R. E. Mitchell B. D. Freeman R. (2003). Diabetic autonomic neuropathy. Diabetes Care 26 (5), 1553–1579. 10.2337/diacare.26.5.1553 12716821

[B172] Walsh J. Griffin B. T. Clarke G. Hyland N. P. (2018). Drug–gut microbiota interactions: implications for neuropharmacology. Br. J. Pharmacol. 175 (24), 4415–4429. 10.1111/bph.14366 29782640 PMC6255959

[B173] Wang X. Xu G. Liu H. Chen Z. Huang S. Yuan J. (2023). Inhibiting apoptosis of schwann cell under the high-glucose condition: a promising approach to treat diabetic peripheral neuropathy using Chinese herbal medicine. Biomed. and Pharmacother. 157, 114059. 10.1016/j.biopha.2022.114059 36462309

[B174] Wang Q. Ye Y. Yang L. Xiao L. Liu J. Zhang W. (2024). Painful diabetic neuropathy: the role of ion channels. Biomed. and Pharmacother. 173, 116417. 10.1016/j.biopha.2024.116417 38490158

[B175] Watanabe T. Sekine S. Naguro I. Sekine Y. Ichijo H. (2015). Apoptosis signal-regulating kinase 1 (ASK1)-p38 pathway-dependent cytoplasmic translocation of the orphan nuclear receptor NR4A2 is required for oxidative stress-induced necrosis. J. Biol. Chem. 290 (17), 10791–10803. 10.1074/jbc.M114.623280 25752609 PMC4409244

[B176] Wazan L. E. I. Widhibrata A. Liu G.-S. (2024). Soluble FLT-1 in angiogenesis: pathophysiological roles and therapeutic implications. Angiogenesis 27 (4), 641–661. 10.1007/s10456-024-09942-8 39207600

[B177] Winter C. D. Iannotti F. Pringle A. K. Trikkas C. Clough G. F. Church M. K. (2002). A microdialysis method for the recovery of IL-1beta, IL-6 and nerve growth factor from human brain *in vivo* . J. Neurosci. Methods 119 (1), 45–50. 10.1016/s0165-0270(02)00153-x 12234634

[B178] Wu R. Liu X. Yin J. Wu H. Cai X. Wang N. (2018). IL-6 receptor blockade ameliorates diabetic nephropathy *via* inhibiting inflammasome in mice. Metabolism 83, 18–24. 10.1016/j.metabol.2018.01.002 29336982

[B179] Xiao Z. Han J. Xiao Q. Huang H. Yang L. Feng J. (2026). Prodrug strategy: molecular design for improving oral drug absorption. Int. J. Pharm. 688, 126459. 10.1016/j.ijpharm.2025.126459 41360171

[B180] Yan S. F. Ramasamy R. Schmidt A. M. (2009). Receptor for AGE (RAGE) and its ligands—cast into leading roles in diabetes and the inflammatory response. J. Mol. Med. 87 (3), 235–247. 10.1007/s00109-009-0439-2 19189073 PMC2659764

[B181] Yang X. Yao W. Shi H. Liu H. Li Y. Gao Y. (2016). Paeoniflorin protects schwann cells against high glucose induced oxidative injury by activating Nrf2/ARE pathway and inhibiting apoptosis. J. Ethnopharmacol. 185, 361–369. 10.1016/j.jep.2016.03.031 26979341

[B182] Yang C. Zhao X. An X. Zhang Y. Sun W. Zhang Y. (2023). Axonal transport deficits in the pathogenesis of diabetic peripheral neuropathy. Front. Endocrinol. 14 1136796, 10.3389/fendo.2023.1136796 37056668 PMC10086245

[B183] Yeung A. M. Huang J. Nguyen K. T. Xu N. Y. Hughes L. T. Agrawal B. K. (2024). Spinal cord stimulation for painful diabetic neuropathy. J. Diabetes Sci. Technol. 18 (1), 168–192. 10.1177/19322968221133795 36384312 PMC10899837

[B184] Yu J.-B. Zhao Z.-X. Peng R. Pan L.-B. Fu J. Ma S.-R. (2019). Gut microbiota-based pharmacokinetics and the antidepressant mechanism of paeoniflorin. Front. Pharmacol. 10 268, 10.3389/fphar.2019.00268 30949054 PMC6435784

[B185] Zang Y. Jiang D. Zhuang X. Chen S. (2023). Changes in the central nervous system in diabetic neuropathy. Heliyon 9 (8), e18368. 10.1016/j.heliyon.2023.e18368 37609411 PMC10440454

[B186] Zhang Z. Tang W. (2018). Drug metabolism in drug discovery and development. Acta Pharm. Sin. B 8 (5), 721–732. 10.1016/j.apsb.2018.04.003 30245961 PMC6146880

[B187] Zhang Y. Li J. Wang T. Wang J. (2014). Amplitude of sensory nerve action potential in early stage diabetic peripheral neuropathy: an analysis of 500 cases. Neural Regen. Res. 9 (14), 1389–1394. 10.4103/1673-5374.137593 25221597 PMC4160871

[B188] Zhang T. Zhu Q. Shao Y. Wang K. Wu Y. (2017). Paeoniflorin prevents TLR2/4-mediated inflammation in type 2 diabetic nephropathy. Biosci. Trends 11 (3), 308–318. 10.5582/bst.2017.01104 28626209

[B189] Zhang D. Wei Y. Chen Y. Chen H. Li J. Yang Y. (2023). Single-nucleus transcriptomic atlas of glial cells in human dorsal root ganglia. Anesthesiol. Perioper. Sci. 1 (3), 17. 10.1007/s44254-023-00015-5

[B190] Zhang X. E. Pang Y. B. Bo Q. Hu S. Y. Xiang J. Y. Yang Z. R. (2023). Protective effect of paeoniflorin in diabetic nephropathy: a preclinical systematic review revealing the mechanism of action. PLoS One 18 (9), e0282275. 10.1371/journal.pone.0282275 37733659 PMC10513216

[B191] Zhang L. Luo Y. L. Xiang Y. Bai X. Y. Qiang R. R. Zhang X. (2024). Ferroptosis inhibitors: past, present and future. Front. Pharmacol. 15, 1407335. 10.3389/fphar.2024.1407335 38846099 PMC11153831

[B192] Zhang Y. Xu L. Huang Y. (2024). Update in the treatment of neuropathic pain, in Translational research in pain and itch *,* eds. Ma C. Huang Y. (Singapore: Springer Nature Singapore), 197–210.

[B193] Zhang L. Wang W. Liu X. Yan K. Li Q. Li M. (2025). Traditional Chinese medicine compounds modulate signaling pathways to improve cardiac-related pathology. Front. Pharmacol. Volume, 16–2025. 10.3389/fphar.2025.1499060 PMC12000890 40242436

[B194] Zhou H. Sun Y. Zhang L. Kang W. Li N. Li Y. (2018). The RhoA/ROCK pathway mediates high glucose-induced cardiomyocyte apoptosis *via* oxidative stress, JNK, and p38MAPK pathways. Diabetes Metab. Res. Rev. 34 (6), e3022. 10.1002/dmrr.3022 29745021

[B195] Zhou Y.-X. Gong X.-H. Zhang H. Peng C. (2020). A review on the pharmacokinetics of paeoniflorin and its anti-inflammatory and immunomodulatory effects. Biomed. and Pharmacother. 130, 110505. 10.1016/j.biopha.2020.110505 32682112

[B196] Zhou R. Yang H. Zhu P. Liu Y. Zhang Y. Zhang W. (2023). Effect of Gut microbiota on the pharmacokinetics of nifedipine in spontaneously hypertensive rats. Pharmaceutics 15 (8), 2085. 10.3390/pharmaceutics15082085 37631299 PMC10458652

[B197] Zhou M. Zhang Y. Shi L. Li L. Zhang D. Gong Z. (2024). Activation and modulation of the AGEs-RAGE axis: implications for inflammatory pathologies and therapeutic interventions – a review. Pharmacol. Res. 206, 107282. 10.1016/j.phrs.2024.107282 38914383

[B198] Zhu J. Hu Z. Luo Y. Liu Y. Luo W. Du X. (2024). Diabetic peripheral neuropathy: pathogenetic mechanisms and treatment. Front. Endocrinol. 14, 1265372. 10.3389/fendo.2023.1265372 38264279 PMC10803883

