# Supplementary material for: Paeoniflorin as a candidate disease-modifying therapy for diabetic peripheral neuropathy: mechanisms, exposure challenges, and translational priorities
Source: Front Pharmacol. 2026 Apr 13;17:1751578. doi: 10.3389/fphar.2026.1751578 (PMC13111209; doi:10.3389/fphar.2026.1751578)
Supplement: Supplementary file 1 [file Table1.docx]

Table S1. Structured appraisal of pharmacological studies investigating paeoniflorin in DPN and mechanistically related models

| Study | Material | Model (in vitro/in vivo) & relevance | Dose range & minimal active | Route & duration | Controls (−/+/disease control) | Key endpoints & results | Quality flags / confidence | Role in review |
| --- | --- | --- | --- | --- | --- | --- | --- | --- |
| Liu et al., 2025 | Purified PF | **In vivo:** STZ-induced diabetic neuropathic pain rats. Relevance: **High (direct DPN pain model).** | PF 50 mg/kg/day (oral gavage) × 4 weeks. Minimal active: NR (single-dose design). | Oral gavage; 4 weeks treatment post-DPN establishment. | Normal control; STZ-DPN disease control; PF-treated group. +drug control: NR. | Mechanical withdrawal threshold ↑; Thermal latency ↑; Gut microbiota composition modulated; SCFAs/metabolic pathways altered; Spinal cord inflammation ↓ (IL-1β/TNF-α ↓); Microglial activation ↓ | Flags: single dose; +control NR; purity NR; no peripheral nerve histology; PK data NR. Confidence: **Moderate for pain-modifying effect; limited disease-modifying structural evidence.** | Direct DPN efficacy |
| Yang et al., 2016 | Purified PF (purchased from National Institute for the Control of Pharmaceutical and Biological Products, Beijing) | **In vitro:** RSC96 Schwann cells + **150 mM glucose** **Relevance:** mechanistic support for DPN Schwann-cell injury. | PF **1/10/100 μM**; minimal active **1 μM** (effects reported across oxidative stress/Nrf2 endpoints). | Cell co-treatment **48 h.** | − 25 mM glucose; disease control 150 mM glucose; +PF groups; positive drug control **NR** | Viability↑; apoptosis↓; ROS/MDA↓; GST/GPX↑; **Nrf2 activation/HO-1, γGCS↑**; Bax/Casp3↓, Bcl-2↑. | Flags: **in vitro only**; +control **NR**; PF purity **NR**; dose–response **Y**. Confidence: **Moderate (mechanistic), not direct DPN efficacy**.a. | DPN-relevant mechanistic |
| Choi et al., 2025 | Purified PF | In vitro: BV-2 microglial cells + LPS (200 ng/mL); In vivo: male C57BL/6J mice; LPS i.p. 0.25 mg/kg. | In vitro PF: 10, 20, 50, 100, 200 μM;  Minimal active: 50 μM;  In vivo PF: 10 or 20 mg/kg. | In vitro: PF pretreatment 1 h; LPS exposure 24 h;  In vivo: PF once daily for 21 days; LPS i.p. once daily for 7 days (days 15–21). | Negative control: saline control (Control group);  Disease control: LPS only (0.25 mg/kg);  Treatment: PF 10 mg/kg + LPS; PF 20 mg/kg + LPS;  Positive control：NR. | In vitro inflammation↓ (NO/iNOS/COX-2; NF-κB/MAPK↓); in vivo behavioral performance↑ with Nrf2/HO-1↑ and neurotrophic signaling↑. | Flags: non-DPN model (relevance limited); positive control NR; PF material definition NR (purity/supplier/batch); blinding/randomization details NR (beyond “random allocation”);  Confidence: Low for DPN translation. | Non-DPN pathway support |
| Chen et al., 2022 | **Decoction (2-herb):** *Paeonia lactiflora* Pall. (root) + *Glycyrrhiza uralensis* Fisch | **In vivo:** male SD rats; **paclitaxel neuropathy (PIPN)**. **Relevance to DPN:** low (different etiology). | SGD **3 vs 6 g/kg** (gavage, day 8–14); minimal active **3 g/kg**. Paclitaxel **2 mg/kg i.p.** on days 1/3/5/7. | Paclitaxel i.p. (days 1–7); SGD **oral gavage daily 7 d** (days 8–14). | − saline/water; disease control paclitaxel-only; +control drug **NR**. | Thermal hyperalgesia↓ (latency↑); DRG/spinal **TRPV1↓, TLR4↓, MyD88↓.** | Flags: **non-DPN**; multi-component; +control **NR**; randomization **Y**; blinding **Y.**  **Confidence: Moderate for PIPN, not DPN evidence.** | Non-DPN pathway support |
| Shao et al., 2019 | Purified **PF**(**purity 98.78% (HPLC)**; supplier **Nanjing GOREN BIO)** | **STZ diabetes ± TLR4−/− (mouse); HG-BMDMs; mechanistic diabetes inflammation, not DPN.** | In vivo: PF 25/50/100 mg/kg i.p. qd ×12 w; in vitro: 10⁻⁸–10⁻³ M screened; **10⁻⁵ M** used for main inhibition assays. | i.p. daily **12 weeks**; macrophage HG assays ~24 h. | WT control; WT+STZ disease control; TLR4−/− mechanistic comparator; +control drug **NR.** | Albuminuria↓; renal CD68↓; iNOS/TNF-α/IL-1β/MCP-1↓; **MyD88/TRIF→NF-κB** signaling ↓. | Flags: **non-DPN endpoints**; +control **NR**; material definition **Y**;randomization/blinding **NR**.  Confidence: **Moderate mechanism**, **not DPN efficacy.** | DPN-relevant mechanistic |
| Song et al., 2017 | Purified **PF** (Sigma; **lot P0038**; **≥98% HPLC**) | **In vitro:** HUVECs + **AOPPs-BSA**. **Relevance to DPN:** supportive for endothelial/VEGF axis, **not nerve/DRG**. | PF **50–400 μM**; **200 μM** selected as strongest (minimal active 50 μM). | PF pretreat **30 min–1 h**; AOPPs **90 min (ROS)** or **24 h** (viability/ATP/MMP/WB). | − DMEM/BSA; disease control AOPPs; mechanistic controls **FPS-ZM1, NAC, Apocynin, BAY11-7082.** | Viability↑; ROS↓; MMP/ATP↑; NF-κB p65↓; **HIF-1α/VEGF↓**; RAGE/Nox2/Nox4↓. | Flags: non-DPN; +control drug **NR**; material definition **Y**; dose–response **Y**. Confidence: **Moderate vascular mechanism**, **not DPN efficacy**. | Non-DPN pathway support |
| Kong et al., 2022 | Purified **PF** (Selleck **S2410**) | In vitro HRCEC under hypoxia; in vivo **OIR** newborn SD rats. **Relevance to DPN:** low. | HRCEC **0.5/5/15 μM** (5 μM significant); OIR rats **100 mg/kg i.p. daily ×14 d.** | In vitro 24–48 h; in vivo i.p. daily **14 d.** | Normoxia control; hypoxia/OIR disease control; +control drug **NR.** | Migration/tube formation↓; **VEGFA↓, HIF-1α↓, p-STAT3↓**; OIR lesions↓. | Flags: non-DPN; +control NR; PF purity/batch NR; dose–response Y. Confidence: **Moderate mechanistic**, **not DPN efficacy**. | Non-DPN pathway support |
| Zhang et al., 2017 | Purified PF (98.78% HPLC; Nanjing GOREN BIO) | **In vivo: db/db (T2DN); in vitro: RAW264.7 + AGEs (200 μg/mL); relevance: diabetic inflammation, not DPN.** | In vivo: 15/30/60 mg/kg i.p.; dose-dependent effects; In vitro: 10⁻⁸–10⁻⁵ M; 10⁻⁵ M used as intervention dose. | In vivo: i.p. daily, 10–12 weeks of age (~2 weeks treatment);In vitro: PF 0.5 h pretreatment + AGEs 24 h. | In vivo: db/m ctrl; db/db model; PF groups. In vitro: ctrl + BSA ctrl + AGEs model; +OxPAPC (TLR2/4 inhibitor); +drug NR. | Albuminuria ↓; TLR2/4, MyD88, p-IRAK1, TRIF, p-IRF3 ↓; NF-κB p65/p-p65 ↓; TNF-α, IL-1β, MCP-1 ↓; Macrophage infiltration ↓. | Flags: non-DPN; +control NR; randomization/blinding NR; dose–response Y; purity Y. Confidence: Moderate (anti-inflammatory mechanism). | DPN-relevant mechanistic |
| Li et al., 2017 | Purified **PF (HPLC ≥98%;** Shanghai Yuanye**)** | **In vivo: SD rat atherosclerosis (Vit D3 + fat emulsion, 15 w); in vitro: VSMCs + PA 100 μM; relevance: vascular inflammation (non-DPN).** | **PF: 10/20 mg/kg p.o. qd ×15 w; in vitro 60/100 μM (5–100 μM screened, 24 h); minimal active: NR.** | **In vivo:** oral/intragastric, **once daily ×15 weeks**. **In vitro:** PF pretreat **1 h** + PA **24 h** stimulation. | **Animals: CG saline; AG AS; PF 10/20 mg/kg; +control simvastatin 5 mg/kg. Cells: control; PA (100 μM) model; PF 60/100 μM; +control simvastatin.** | **In vivo: TC/TG/LDL-C↓; aortic lesions↓; IL-1β/IL-6/TNF-α↓. Mechanism (aorta+VSMCs): TLR4↓, MyD88↓, p-IκBα↓, p-NF-κB p65↓.** | Flags: **non-DPN model**; minimal active dose/concentration **NR.** **Confidence: Moderate for TLR4/MyD88/NF-κB anti-inflammatory mechanism; Low for direct DPN efficacy (not a neuropathy model).** | Non-DPN pathway support |

Table S1 Structured critical appraisal of original pharmacological studies evaluating paeoniflorin (PF) in diabetic peripheral neuropathy (DPN) models and mechanistically related disease contexts. Extracted parameters include material definition, model type (in vitro/in vivo), dose range and minimal active concentration (where available), route and duration, control conditions, primary endpoints, and reporting completeness.
Abbreviations: Y, yes (clearly reported); NR, not reported.
